# Developmental atlas of the indirect-developing sea urchin *Paracentrotus lividus*: From fertilization to juvenile stages

**DOI:** 10.3389/fcell.2022.966408

**Published:** 2022-10-31

**Authors:** Laurent Formery, Axel Wakefield, Maeva Gesson, Ludovic Toisoul, Guy Lhomond, Laurent Gilletta, Régis Lasbleiz, Michael Schubert, Jenifer C. Croce

**Affiliations:** ^1^ Sorbonne Université, CNRS, Institut de la Mer de Villefranche (IMEV), Laboratoire de Biologie du Développement de Villefranche-sur-Mer (LBDV), Evolution of Intercellular Signaling in Development (EvoInSiDe), Villefranche-sur-Mer, France; ^2^ Sorbonne Université, CNRS, Institut de la Mer de Villefranche (IMEV), Laboratoire de Biologie du Développement de Villefranche-sur-Mer (LBDV), Villefranche-sur-Mer, France; ^3^ Sorbonne Université, CNRS, Institut de la Mer de Villefranche (IMEV), Service Aquariologie du Centre de Ressources de Biologie Marine (CRBM), Villefranche-sur-Mer, France

**Keywords:** echinoderm, staging scheme, larva, coelom, rudiment, metamorphosis

## Abstract

The sea urchin *Paracentrotus lividus* has been used as a model system in biology for more than a century. Over the past decades, it has been at the center of a number of studies in cell, developmental, ecological, toxicological, evolutionary, and aquaculture research. Due to this previous work, a significant amount of information is already available on the development of this species. However, this information is fragmented and rather incomplete. Here, we propose a comprehensive developmental atlas for this sea urchin species*,* describing its ontogeny from fertilization to juvenile stages. Our staging scheme includes three periods divided into 33 stages, plus 15 independent stages focused on the development of the coeloms and the adult rudiment. For each stage, we provide a thorough description based on observations made on live specimens using light microscopy, and when needed on fixed specimens using confocal microscopy. Our descriptions include, for each stage, the main anatomical characteristics related, for instance, to cell division, tissue morphogenesis, and/or organogenesis. Altogether, this work is the first of its kind providing, in a single study, a comprehensive description of the development of *P. lividus* embryos, larvae, and juveniles, including details on skeletogenesis, ciliogenesis, myogenesis, coelomogenesis, and formation of the adult rudiment as well as on the process of metamorphosis in live specimens. Given the renewed interest for the use of sea urchins in ecotoxicological, developmental, and evolutionary studies as well as in using marine invertebrates as alternative model systems for biomedical investigations, this study will greatly benefit the scientific community and will serve as a reference for specialists and non-specialists interested in studying sea urchins.

## Introduction

**FIGURE 1:**
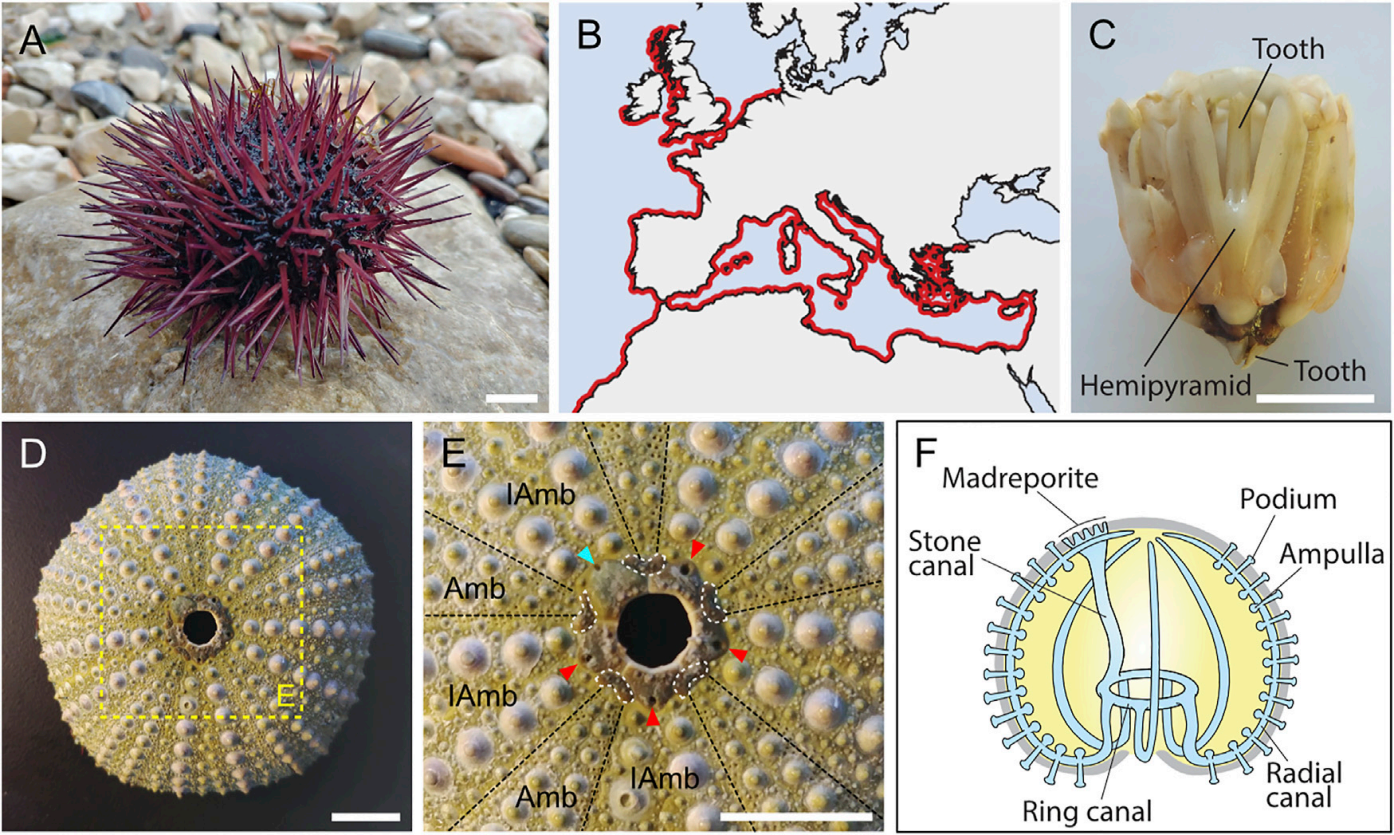
General information on *Paracentrotus lividus* adults. **(A)** Photography of an adult specimen of the sea urchin species *P. lividus*. In the wild, *P. lividus* adults can be purple (as shown here), green, or brown. **(B)** Schematic representation of the geographical distribution of *P. lividus*, based on the Ocean Biodiversity Information System (OBIS, 2021). **(C)** Photography of a dissected masticatory apparatus of a *P. lividus* adult (i.e., Aristotle’s lantern). **(D)** Photography of the calcitic endoskeleton of a *P. lividus* adult. No matter the outer color of the adult, its endoskeleton is always green, more or less pale. **(E)** Close-up of the aboral surface and the central disk of the calcitic endoskeleton of a *P. lividus* adult, corresponding to the region highlighted by the yellow box in **(D)**. In **(E)**, note that the endoskeleton is composed of five ambulacra separated from each other by five interambulacra. Note further that each of the five gonopores (pores through which the gametes are released) is held by a genital plate (marked by the cyan and red arrowheads). One of the five genital plates is bigger than the others, it is the madreporite (marked by the cyan arrowhead), which corresponds to a sieve plate enabling water to enter the water vascular system. In addition, each genital plate is interconnected by five ocular plates (delineated by the white dotted lines), which together form the central disk of the aboral surface. The hole, in the middle of the central disk, does not correspond to the anus of the animal. In live animals, this hole is filled by periproct plates, which are not attached to the rest of the endoskeleton and are thus rarely conserved on an endoskeleton stripped of the ‘living parts’. The anus itself is almost impossible to see when the periproct system is intact. **(F)** Schematics of the echinoid water vascular system. The water vascular system is composed of a stone canal connected, on one side, to the madreporite, and, on the other side, to the ring canal. The ring canal is itself connected to five radial canals that are connected to ampullae and podia (or tube feet). Circulation of water through the water vascular system enables the podia to extend and retract, allowing the animal to move on the substrate. Scale bar: **(A,C–E)** 1 cm. Amb: ambulacra, IAmb: interambulacra.

**FIGURE 2:**
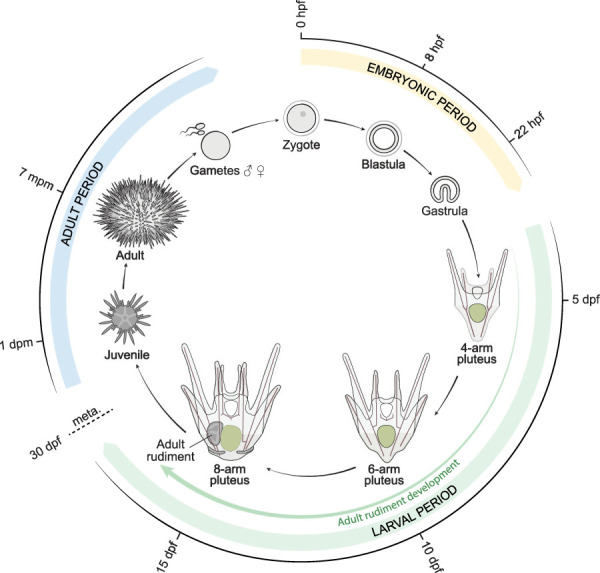
Schematic representation of the life cycle of the sea urchin *Paracentrotus lividus*. Approximate timing for each of the represented stages is provided based on our rearing conditions at 18°C. dpf: days post-fertilization; dpm: days post-metamorphosis; hpf: hours post-fertilization; meta.: metamorphosis; mpm: months post-metamorphosis.

The common sea urchin (or echinoid) *Paracentrotus lividus* (Figure 1A) (Lamarck, 1816) is a Mediterranean and Atlantic species (Figure 1B), which is particularly common in Western Europe and North Africa (Ouréns et al., 2011). It is usually found in shallow coastal waters and living on rocky substrates or in seagrass meadows (Boudouresque and Verlaque, 2013). Phylogenetically, *P. lividus* belongs to the phylum Echinodermata (Cannon et al., 2014; Telford et al., 2014). As such, it is characterized anatomically, in its adult form, by four echinoderm synapomorphies: a calcite endoskeleton, a mutable collagenous tissue, a water vascular system, and a pentaradial symmetry (Hyman, 1955; Nichols, 1972; Wilkie, 2002; Smith, 2008). In addition, as an echinoid, *P. lividus* is anatomically distinguishable from other echinoderms 1) by the presence of a complex masticatory apparatus called Aristotle’s lantern (Figure 1C) and 2) by a rigid endoskeleton made of tightly jointed skeletal plates forming a hard shell (or test) (Figure 1D), which protects the internal organs and bears articulated spines. In echinoids, the pentaradial symmetry of the animal is further obvious, chiefly, at the level of the endoskeleton and of the water vascular system (Figures 1E,F). The endoskeleton is characterized by ten discernable fields, i.e., five ambulacra separated by five interambulacra (Figure 1E). The water vascular system, which consists of an internal network of fluid-filled canals, has five radial canals, each one spreading along one of the five ambulacral fields and protruding outside the test through multiple podia (or tube feet) (Figure 1F) (Nichols, 1972).

As all other echinoids, *P. lividus* animals are gonochoric. This means that even though males and females are indiscernible externally, each individual produces only one of the two types of gametes, eggs or spermatozoids (Gianguzza et al., 2009). Spawning takes place in the water column during coordinated events, and fertilization is external, hence making this species easily amenable for *in vitro* fertilization in the laboratory (Tenuzzo et al., 2012; Ortiz et al., 2019). Following fertilization, like many other echinoids, *P. lividus* exhibits an indirect mode of development (Cellario and Fenaux, 1990; Gosselin and Jangoux, 1998). Thus, the animal undergoes a bi-phasic lifecycle with the initial formation, following embryogenesis, of a pelagic, planktotrophic larva called the echinopluteus (or pluteus) larva that exhibits a classical bilateral symmetry. The pentaradial adult body plan forms only later, within the larva, on the left side of the digestive tract, as a vesicle called the rudiment. Once the rudiment is fully formed, the larva has reached competency, and, upon adequate environmental cues, it will undergo a dramatic morphological reorganization, called metamorphosis. During metamorphosis, the planktonic, bilateral larva thus transforms into a benthic, pentaradial juvenile. The juvenile corresponds to a miniature version of the adult and, upon grazing on algae, it will eventually grow in size and develop reproductive organs.

Over the past century, the interest in the sea urchin species *P. lividus* has been substantial, not only because of its ecological and economic significance, but also because it is a suitable model system for biological and biomedical research (McClay, 2011; Bernardo and Carlo, 2017). Indeed, over the past years, *P. lividus* has been an important model organism in ecotoxicological and climate change surveys (Aluigi et al., 2008; Dorey et al., 2018). From a biological and biomedical point of view, *P. lividus* has further contributed to a number of important discoveries. Most importantly, it enabled the demonstration of the concepts of regulative development (Driesch, 1892), of chromosomal inheritance (Boveri, 1902), and of inductive interaction (reviewed in Hörstadius, 1973). In addition, based on the use of modern gene manipulation approaches, it provided important insights into developmental gene regulatory networks, patterning mechanisms, and morphogenesis (e.g., Saudemont et al., 2010; Lhomond et al., 2012; Gildor et al., 2016). On a different note, the gonads of *P. lividus* are further considered a delicacy and luxury seafood in many Mediterranean countries, making *P. lividus* adults a valuable resource with important commercial value (Matsiori et al., 2012; Baião et al., 2021) and a target for aquaculture-related research (Castilla-Gavilán et al., 2018).

Due to both the scientific and commercial importance of *P. lividus*, several studies have already reported the different phases of the life cycle of this sea urchin species. For instance, classical developmental descriptions of embryos and larvae have been published by Boveri (1901), von Ubisch (1913), and Hörstadius (1973). Likewise, the transition from the larval to the adult stage of *P. lividus*, i.e., from competent larva to early juvenile, has been reported by Gosselin and Jangoux (1998). A staging scheme for early juvenile stages of *P. lividus*, i.e., during the first 4 weeks of its post-metamorphic life, has also recently been provided by Thompson et al. (2021). Similarly, the adult stage of *P. lividus* has been carefully studied over the past decades by several zoologists interested in muscular or skeletal anatomy (Stauber, 1993; Wilkie et al., 1998; Ziegler et al., 2012), pigmentation (Goodwin and Srisukh, 1950), and calcification (Ameye et al., 1999; Politi et al., 2004). Despite these previous investigations, some aspects of *P. lividus* development remain largely unknown. These include, for instance, the development of the coeloms and the rudiment within the larva as well as the development of the ciliated structures and muscles during the embryonic and larval periods.

Our goal here is to describe the aspects of *P. lividus* development that have so far been neglected and to provide a rigorous and exhaustive staging scheme for this sea urchin species from fertilization to post-metamorphic juvenile stages. For all stages, even those previously described, we provide a detailed and comprehensive morphological description, using images recorded on live specimens. In addition, we report the details of ciliogenesis and muscle development based on immunohistochemistry assays. Altogether, this study hence offers a valuable and complete reference for all aspects of *P. lividus* development, including an analysis, in live specimens, of the process of metamorphosis. Over the past decades, the scientific community has been looking for alternative model systems with experimental amenability and relatively short life-cycles. *P. lividus* is certainly a suitable candidate, with existing genomic and transcriptomic resources, working protocols for functional analyses, and sexual maturity reached in 6–8 months. The present work hence represents an additional tool for future research using *P. lividus* as a model system. It provides an updated staging scheme for *P. lividus* that the scientific community will be able to rely on for future research in this species, allowing standardized and comparable surveys to be carried out across different laboratories. Given the level of detail provided in this study, the stage descriptions for *P. lividus* development can further serve as a valuable basis for detailed comparisons with other echinoid species.

## Materials and methods

### Animal husbandry

Adults *Paracentrotus lividus* were collected in the bay of Villefranche-sur-Mer (France). Gamete collection was performed as previously described (Lepage and Gache, 1989). For fertilization, 10 µL of sperm was diluted in 1 ml of sea water. The sperm dilution was validated by eye, by estimating the turbidity of the sea water. The dilution was added to a beaker containing unfertilized eggs in about 150 ml of sea water and a manual stirring was applied to homogenize the distribution of sperm. After adding the sperm, the success of fertilization, marked by the elevation of the fertilization envelope, was immediately monitored under a binocular, to make sure that the sperm concentration added was sufficient to enable fertilization but low enough to avoid triggering polyspermy. Fertilized eggs were then rinsed twice to remove the excess of sperm. After settlement of the eggs at the bottom of the beaker, about 120 ml of sea water were thus replaced each time. Embryos and larvae were then cultured, under constant mechanical stirring, at 18°C in natural seawater collected at “point B” in the bay of Villefranche-sur-Mer (43°41 N 07°19 E) at a depth of 200 m and filtered using a 0.2 µm mesh. Following fertilization, embryos were cultured at a density of 100 embryos per mL until 48 h post-fertilization (hpf). At 48 hpf, the culture concentration was adjusted to 1 larva per mL. From 48 hpf on, water exchange and feeding were performed as previously reported (Formery et al., 2021), except that the larvae were fed, every week day, with freshly grown cultures of the microalgae *Dunaliella salina* (strain MCCV127) and *Rhodomonas salina* (strain MCCV118). After about 30 days post-fertilization (dpf), the larvae started undergoing spontaneous metamorphosis in the culture vessels. However, to document this event, synchronous metamorphosis of competent larvae was triggered by adding Dibromomethane (Sigma-Aldrich, Saint-Quentin-Fallavier, France), in the culture vessels, at a final concentration of 1.25 mg/ml. Dibromomethane is a halogenated aliphatic compound secreted by red coralline algae that has been reported as a chemical inducer of larval metamorphosis for several marine species (Agatsuma et al., 2006; Taris et al., 2010). Upon metamorphosis, the juveniles were cultured at 16°C, under constant seawater flow, with the water being pumped at a depth of 5 m, decanted, and cooled down to 16–18°C. Starting 7 days post-metamorphosis (dpm), the juveniles were fed with rehydrated *Tetraselmis suecica* algae (Inalve, Villefranche-sur-Mer, France).

### Anatomical observation (light microscopy)

*P. lividus* development, from fertilization to juvenile stages, was recorded on specimens mounted alive. *P. lividus* embryos, larvae, and juveniles were imaged using a Zeiss A2 (Axio Imager 2) light microscope (Zeiss, Jena, Germany), under either bright-field, differential interference contrast (DIC), or polarized light. *P. lividus* larvae were also imaged, under dark-field, using a Zeiss SteREO Discovery V20 microscope (Zeiss, Jena, Germany). Metamorphosis was imaged using a Zeiss Axio Observer Z1 microscope (Zeiss, Jena, Germany). In all cases, images were captured using the Zen software suite (Zeiss, Jena, Germany). To record embryos, larvae, and juveniles, specimens were mounted in 0.2 µm filtered seawater, between a slide and a cover slip, using clay to generate spacers and enable orientation of the specimens. Specimens were mounted alive, except, in some cases, for the larvae between the *4-arm* and *8-arm pluteus stages*. At these stages, larvae were immobilized, when needed, before mounting and imaging, to prevent them from swimming. For this, a drop of 8% paraformaldehyde (#P6148, Sigma-Aldrich, Saint-Quentin-Fallavier, France) prepared in sea water was added to a small Petri dish containing the larvae in filtered sea water. Multiple z-stacks were taken manually when required. To record metamorphosis, a multi-well agarose gel was generated using a 3D-printed mold that was deposited within a Petri dish containing a 1% agarose gel prepared in 0.2 µm filtered seawater. The mold was designed with conical spikes to create well diameters from 1.5 mm at the bottom to 5 mm at the top. In addition, the spikes were generated with an angle lower than the numerical aperture of the objectives used on the microscope to avoid light reflection. Competent larvae were placed in the agarose wells individually, with 0.2 µm filtered seawater filling the wells. Images were acquired using a Marzhauser SCAN IM (Inverse Microscopes) 130 × 10 motorized stage (Marzhauser, Wetzlar, Germany), allowing acquisitions in multi-position. Images were further acquired in z-stacks with a 20 µm step every 1 min and for about 2 h. For all images, projections were generated, when needed, using the focus merging option of Affinity Photo (Serif, Nottingham, United Kingdom). Images were further processed using Adobe Photoshop (Adobe Inc., San Jose, United States) or Affinity Photo (Serif, Nottingham, United Kingdom), and scale bars were added using ImageJ version 1.44o (Schneider et al., 2012), which was also used to generate the movie of metamorphosis. All figures were formatted using Adobe Illustrator (Adobe Inc., San Jose, United States) or Affinity Designer (Serif, Nottingham, United Kingdom).

### Immunohistochemistry (confocal microscopy) and *in situ* hybridization

Immunohistochemistry assays were performed as previously described in Formery et al. (2021). The primary antibody used was a mouse anti-acetylated α-tubulin (#T6793, Sigma-Aldrich, Saint-Quentin-Fallavier, France) prepared at 1:200 in PBST or in PBST plus 0.005 U/μl rhodamine phalloidin (#R415, Thermo Fisher Scientific, Illkirch-Graffenstaden, France) to label F-actin particularly enriched in muscles. The secondary antibody used was an Alexa Fluor 647 goat anti-mouse IgG H&L (#ab150115, Abcam, Cambridge, United Kingdom) diluted at 1:200 in PBST. In all specimens, nuclear staining was also performed following the method of Formery et al. (2021), except that we used either TO-PRO1 Iodide (515/531) or TO-PRO3 Iodide (642/661) (#T3602 or #T3605, Thermo Fisher Scientific, Illkirch-Graffenstaden, France), depending on the fluorochrome needed. Observation and imaging of the labeled specimens were carried out using a Leica SP8 confocal microscope (Leica, Wetzlar, Germany). For each sample, series of optical sections were taken at a z-step interval of 1–2 μm and multichannel acquisitions were obtained by sequential imaging. Confocal optical sections were compiled into maximum intensity z-projections using ImageJ version 1.44o (Schneider et al., 2012), and scale bars were added using the same software.

*In situ* hybridization assays were carried out as previously described in Robert et al. (2014). The riboprobe used for the *P. lividus myosin heavy chain* (*mhc*) gene corresponded to a 2066 base pair-long partial cDNA (GenBank accession number: OM307457). The probe was used at a final concentration of 1 ng/μL, and images were acquired using a Zeiss A2 (Axio Imager 2) microscope (Zeiss, Jena, Germany) under differential interference contrast (DIC) light. Scale bars were added using ImageJ version 1.44o (Schneider et al., 2012). For immunohistochemistry, as for *in situ* hybridization, images were processed using either Adobe Photoshop (Adobe Inc., San Jose, United States) or Affinity Photo (Serif, Nottingham, United Kingdom), and figures were compiled using either Adobe Illustrator (Adobe Inc., San Jose, United States) or Affinity Designer (Serif, Nottingham, United Kingdom).

## Results

### *Paracentrotus lividus* development: Staging scheme overview and nomenclature

**FIGURE 3:**
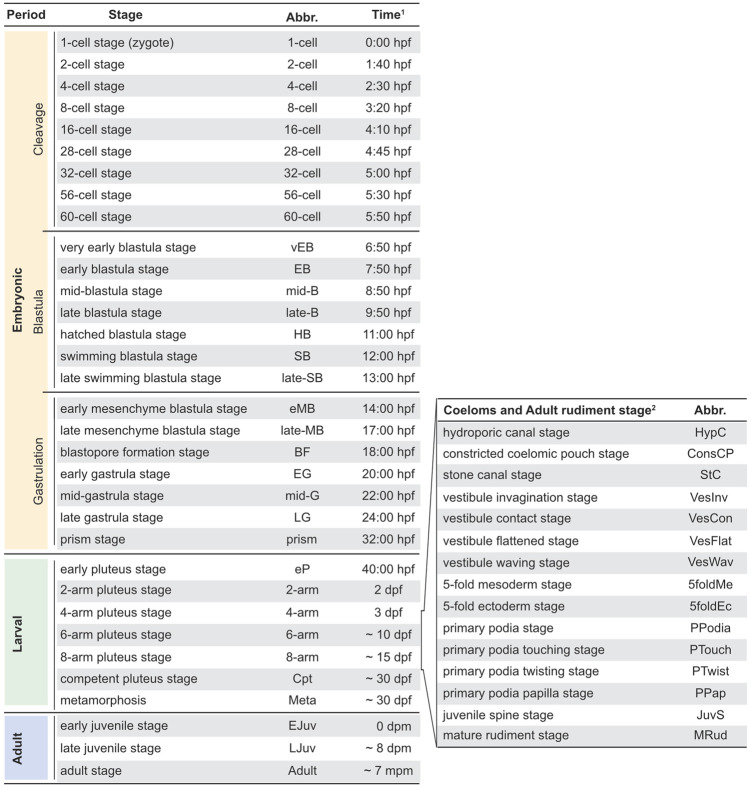
*Paracentrotus lividus* staging scheme. In the left panel, timing ^(1)^ is provided for our rearing conditions at 18°C. Note that a certain degree of heterochrony was observed during larval and adult periods ( ~ ). As development of the coeloms and the adult rudiment proceeded in parallel to that of the larva, during the 4-, 6-, and 8-arm pluteus stages, a subsection for coeloms and adult rudiment development is provided in the right panel. ^(2)^ Due to the heterochrony of the developmental trajectories of the coeloms and the adult rudiment, even within a given culture, a developmental timing for these events cannot be provided. Abbr.: abbreviation; dpf: days post-fertilization; dpm: days post-metamorphosis; hpf: hours post-fertilization; mpm: months post-metamorphosis.

The development of *P. lividus*, observed under our rearing conditions from egg to post-metamorphic juveniles, occurred as depicted in Figures 2, 3. We defined each developmental stage based on ontogenic features, which were readily detectable in whole-mount specimens using regular transmitted light microscopy and which we will describe in detail in the subsequent sections. We subdivided the development of *P. lividus* in three main periods: the embryonic period (or embryogenesis), the larval period, and the adult period (Figures 2, 3). The embryonic period covers the development of the embryo from fertilization to the *prism stage*, right before the opening of the larval mouth and the establishment of the larval body plan. The larval period subsequently covers the larval growth and formation of the adult rudiment within the larva. This period starts upon the opening of the larval mouth, at the so-called *early pluteus stage*, and ends with metamorphosis. The adult period finally covers the growth of the juvenile and the acquisition of sexual maturity. This period starts right after metamorphosis, with the formation of the benthic, pentaradial juvenile and ends with the death of the animal, which has been estimated to occur after 6–9 years for wild *P. lividus* adults (Crapp and Willis, 1975). Each of the defined developmental periods was then subdivided into several distinct developmental stages, which cover various time spans (Figure 3). The stages during the embryonic and larval periods as well as for rudiment and juvenile development were defined based on previous descriptions made for *P. lividus* and other sea urchin species (Hörstadius, 1973; Okazaki, 1975; Gosselin and Jangoux, 1998; Smith, 2008; Heyland and Hodin, 2014).

After recording the development of thousands of embryos and larvae, from more than thirty independent cultures, we found that the timing of development, during the embryonic period, was stereotypical (i.e., similar within and between cultures). By contrast, during the larval and adult periods, a certain degree of variation was observed, even within a given culture, and despite the fact that the larvae and juveniles were healthy throughout the culture period. With time, and even though the larvae were fed *ad libidum*, differences were observed between larvae, chiefly due to a heterochrony of ontogenic events, which is consistent however with previous reports (Strathmann et al., 1992; Carrier et al., 2015). For instance, we observed that the duration of the larval period varied between 4 and 7 weeks, that rudiment formation started between 7 and 14 days post-fertilization, and that spontaneous metamorphosis occurred between 1 day and 3 weeks after acquisition of competency. The most significant and recurrent heterochrony observed was between the developmental trajectories of the larva and the adult rudiment. The ontogeny of certain larval structures, such as the larval arms and the epaulettes, were often mismatched with the growth of the coeloms and the adult rudiment, and this even if development of the larval structures, on one hand, and of the coeloms and adult rudiment, on the other hand, followed their respective, stereotypical developmental paths. In Figure 3, we thus propose a separate set of staging schemes for the development of the coeloms and the adult rudiment, which corresponds to a subset of the larval period (Figure 3). This subset usually started during the *4-arm pluteus stage* and always ended during the *8-arm pluteus stage*. In addition, although we provide an approximate timing for the main stages of the embryonic, larval, and adult periods (Figure 3), due to the heterochronic nature of their development, we decided not to include such a timing for the stages related to the formation of the coeloms and the adult rudiment. Instead, we named these stages according to morphological traits that were easily identifiable (Figure 3).

It should be noted, furthermore, that different terms have been used in previous studies to identify the main body axes of echinoid embryos, larvae, and juveniles (e.g., Hyman, 1955; Okazaki, 1975; Gosselin and Jangoux, 1998; Smith et al., 2008; Range et al., 2013). Here, the set of terms that will be used is illustrated in Supplementary Figure S1. For embryos, we chose to refer to the primary body axis as the animal-vegetal axis, with the vegetal pole being marked by the presence of the micromeres and then the blastopore. For larvae, we identified the axes following their ontogenetic appearance during embryonic development, meaning that we considered that the anus marks the posterior pole. We further considered that the larval arms develop on the ventral side of the larvae, corresponding to the side bearing the mouth. Consequently, the larval apex forms on the dorsal side. For juveniles and adults, we finally referred to the side bearing the adult mouth as the oral side, with the side bearing the anus thus being the aboral side. To define the pentaradial symmetry of the adult body, already distinguishable in the rudiment, we chose to use the nomenclature proposed by Carpenter (Carpenter, 1884). In this nomenclature, the five rays correspond to the position of the ambulacra and they are respectively called ambulacrum A, B, C, D, and E, with ambulacrum A facing the madreporite, a specific skeletal piece of the central disk on the aboral surface (Figure 1E, Supplementary Figure S1). In addition, these letters are positioned counterclockwise when the juvenile is looked at from the aboral side, and clockwise when it is looked at from the oral side (Carpenter, 1884; Paul and Hotchkiss, 2020) (Supplementary Figure S1). Accordingly, the interambulacra are named AB, BC, CD, DE, and EA, respectively, with the madreporite being located in interambulacrum CD (Supplementary Figure S1).

### Embryonic development

#### Spawning and fertilization

**FIGURE 4:**
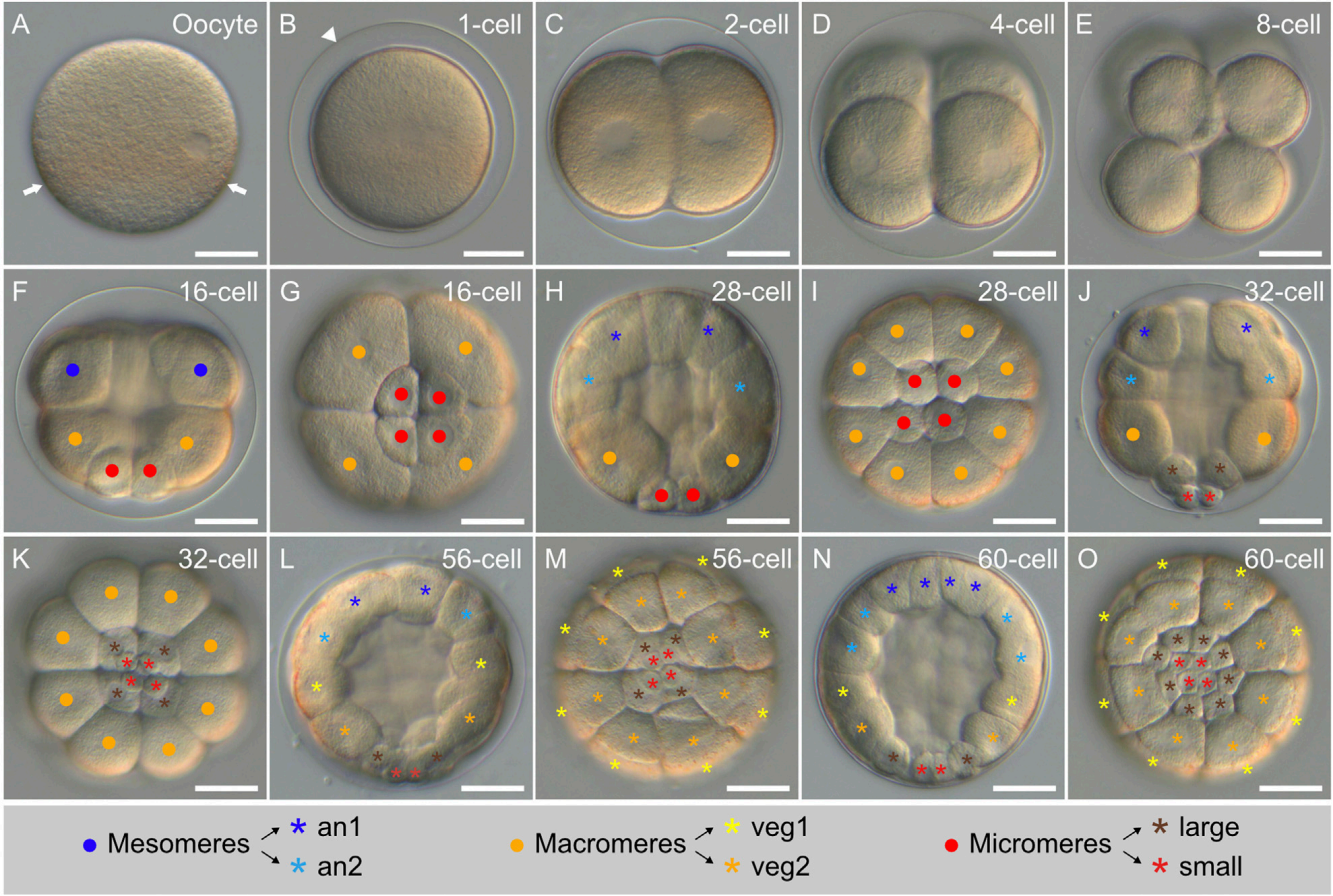
Early cleavage stages of *Paracentrotus lividus* under light microscopy. Developmental stages are as follows: **(A)** unfertilized egg (oocyte); **(B)** zygote (or 1-cell stage) (1-cell); **(C)** 2-cell stage (2-cell); **(D)** 4-cell stage (4-cell); **(E)** 8-cell stage (8-cell); **(F,G)** 16-cell stage (16-cell); **(H,I)** 28-cell stage (28-cell); **(J,K)** 32-cell stage (32-cell); **(L,M)** 56-cell stage (56-cell); **(N,O)** 60-cell stage (60-cell). In **(A–F,H,J,L,N)**, the embryos are in lateral view with the animal pole up. In **(G,I,K,M,O)**, the embryos are in vegetal view. In **(A)**, arrows highlight the equatorial pigment band. In **(B)**, the arrowhead marks the fertilization envelope. In **(F–K)**, dots in blue, orange, and red respectively indicate: the mesomeres, the macromeres, and the micromeres. In **(H–O)**, asterisks in dark blue, light blue, yellow, orange, brown, and red respectively mark: the an1, an2, veg1, and veg2 cells as well as the large and the small micromeres. The schematic legend below the images illustrates cell lineage relationships. Scale bar: **(A–O)** 30 μm. an: animal; veg: vegetal.

Sexually mature adult males and females of *P. lividus* displayed 5 gonopores (see Figure 1E). During spawning, spermatozoids and oocytes were released through the gonopores by the males and by the females, respectively. Freshly-spawned *P. lividus* oocytes had a size of about 90 µm (Figure 4A). They were spherical and mostly transparent, with a conspicuous band of orange-pigmented granules in a subequatorial position, which remained visible during subsequent developmental stages (Figure 4). In the wild, *P. lividus* fertilization takes place externally and can thus easily be reproduced in the laboratory (Foltz et al., 2004). Entry of a spermatozoid into an oocyte triggers, in the latter, the immediate separation of the vitelline envelope from the surface of the oocyte. Accordingly, within seconds after spermatozoid addition, we observed inflation of the vitelline envelopes. This led to the appearance of a thin translucent membrane, called the fertilization envelope, which surrounded the *zygote* (or *1-cell stage* embryo) (Figure 4B). Fertilizations were extremely synchronous, with thousands of oocytes fertilized simultaneously.

#### Cleavage stages

Following fertilization, the *zygotes* underwent first cleavage after about 1 h and 40 min (1:40 h post-fertilization - 1:40 hpf), when reared at 18°C. This cleavage was holoblastic and meridional. It gave rise to a *2-cell stage* embryo, composed of two blastomeres, separated by a plane parallel to the animal-vegetal axis (Figure 4C). The second cleavage occurred 2:30 hpf and resulted in a *4-cell stage* embryo (Figure 4D). This cleavage was also holoblastic and meridional, yet perpendicular to the first one. At the *4-cell stage*, the embryos were thus composed of four blastomeres of equal size, each containing an equal volume of both animal and vegetal cytoplasm. The third cleavage took place 3:20 hpf and resulted in an *8-cell stage* embryo (Figure 4E). This cleavage was equatorial, i.e., perpendicular to the two first ones. It separated four animal from four vegetal blastomeres, generating two territories with different cytoplasmic contents: the animal and the vegetal territories. The fourth cleavage subsequently occurred at 4:10 hpf and resulted in a *16-cell stage* embryo (Figures 4F,G). At this stage, cell division patterns became different between the animal and the vegetal hemispheres as well as unequal in the vegetal hemisphere. Thus, in the animal hemisphere, the fourth cleavage was meridional, generating a ring of eight blastomeres of equal size (referred to as the mesomeres). In the vegetal hemisphere, the fourth cleavage was equatorial and unequal, producing four large cells (referred to as the macromeres) positioned right below the mesomeres and four small cells (referred to as the micromeres) marking the vegetal pole.

Following fourth cleavage, cell divisions became furthermore asynchronous, with the mesomeres and the macromeres dividing prior to the micromeres. Fifth cleavage started in the mesomeres and macromeres at 4:45 hpf, giving rise to an initial, intermediate *28-cell stage* embryo (Figures 4H,I). The eight mesomeres divided equatorially and equally, producing two cell tiers of eight cells each: the animal tier 1 (an1), marking the animal pole, and the animal tier 2 (an2), located below an1. The macromeres divided meridionally and equally, generating a single ring of eight cells of equal volume located below an2. After 15 min, the micromeres also underwent their fifth cleavage, leading to a *32-cell stage* embryo (Figures 4J,K). The micromeres divided equatorially and unequally, producing four large micromeres (just below the macromeres) and four small micromeres (marking the vegetal pole).

Sixth cleavage took place in the mesomeres and the macromeres at 5:30 hpf and resulted in an intermediate *56-cell stage* embryo (Figures 4L,M). During this cleavage, the cells of the an1 and an2 tiers divided equally and meridionally, producing a new an1 and a new an2 tier now composed of 16 cells each. The macromeres underwent an equal but equatorial cleavage, thereby leading to the emergence of two vegetal tiers, each composed of eight cells: the vegetal tier 1 (veg1), located below an2, and the vegetal tier 2 (veg2), located below veg1. 20 min later, at 5:50 hpf, the large micromeres underwent their sixth cleavage, which was meridional and equal. This thus generated a single ring of eight micromeres of equal volume, with the small micromeres at their center not displaying any sign of cell division (Figures 4N,O). By 6:00 hpf, the embryos were thus at the *60-cell stage* and composed, from the animal to the vegetal pole, of sixteen an1 cells, sixteen an2 cells, eight veg1 cells, eight veg2 cells, eight large micromeres, and four small micromeres (Figures 4N,O).

#### Blastula stages

**FIGURE 5:**
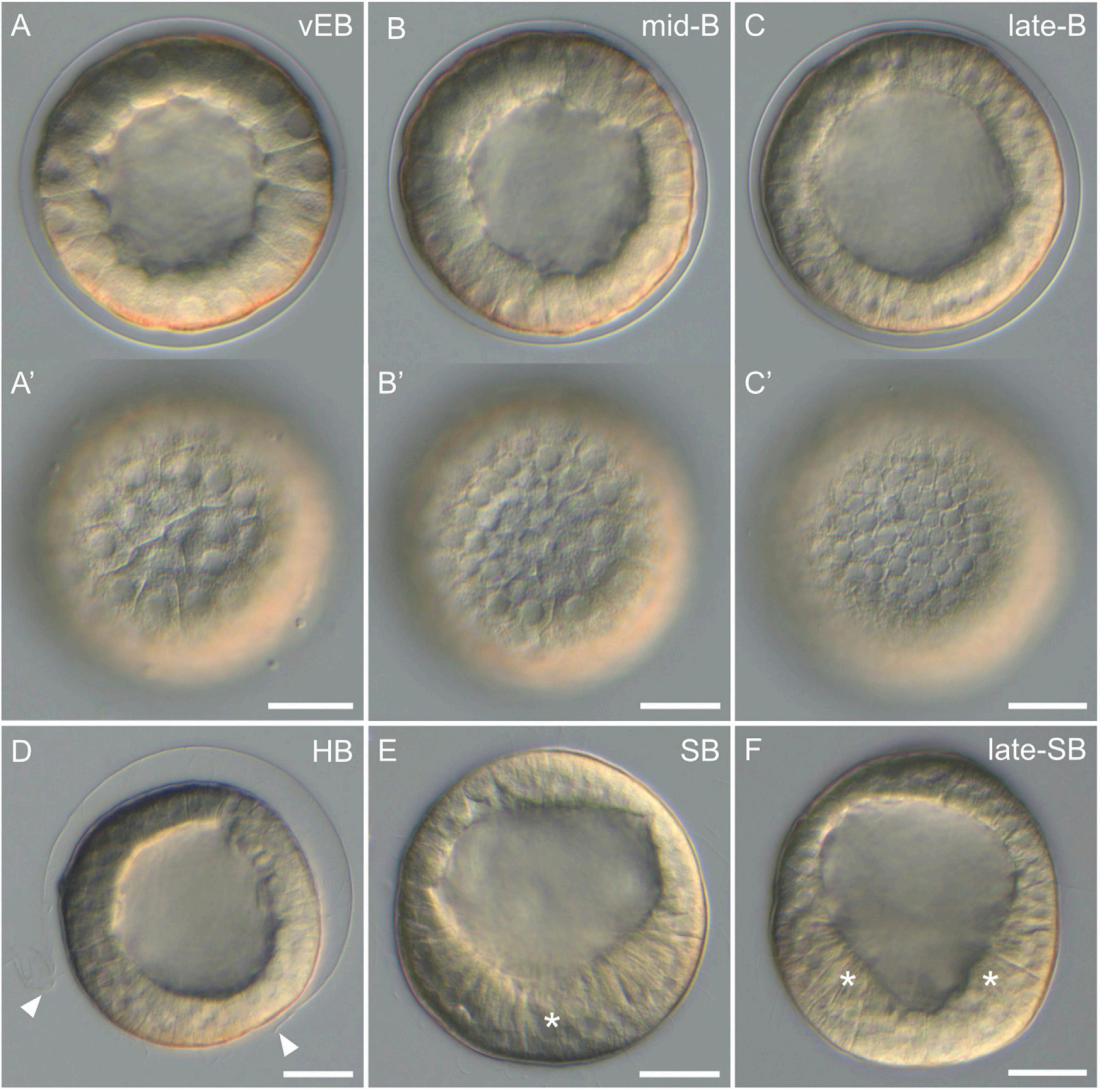
Blastula stages of *Paracentrotus lividus* under light microscopy. Developmental stages are as follows: **(A,A')** very early blastula stage (vEB); **(B,B')** mid-blastula stage (mid-B); **(C,C')** late blastula stage (late-B); **(D)** hatched blastula stage (HB); **(E)** swimming blastula stage (SB); **(F)** late swimming blastula stage (late-SB). In **(A–F)**, the embryos are in lateral view with the animal pole up. **(A',B',C′)** are optical surface views of, respectively, **(A–C)**. In **(D)**, arrowheads mark the rupture of the fertilization envelope. In **(E)**, the asterisk indicates the thickening of the cells constituting the vegetal plate, at the vegetal pole. In **(F)**, asterisks highlight the “V” shape of the vegetal plate. Scale bar: **(A–F)** 30 µm.

After the *60-cell stage*, cell divisions continued, but the embryos stopped displaying any visual landmark to differentiate the animal-vegetal axis, the distinct cell tiers, or the different developmental stages. As such, the embryos were now referred to as blastulae, and we observed and imaged them every hour after the *60-cell stage*. Starting at the *very early blastula stage* (1 h after the *60-cell stage*), the embryos, still enclosed in the fertilization envelope, exhibited a characteristic spherical organization (Figure 5A). The cells formed a monolayered, hollow sphere that surrounded a central, fluid-filled cavity called the blastocoel. During the subsequent stages, i.e., the *early blastula stage* (2 h after the *60-cell stage*), the *mid-blastula stage* (3 h after the *60-cell stage*), and the *late blastula stage* (4 h after the *60-cell stage*), the embryos were still enclosed in the fertilization envelope and were morphologically very similar to each other. They exhibited a spherical shape composed of a monolayer of cells surrounding the blastocoel (Figures 5B,C). Yet, during these stages, and without affecting the overall size of the embryos, the cells continued to divide and became smaller (Figures 5A'–C'), thereby progressively thinning the wall of the blastulae (Figures 5A–C). Consequently, the blastocoel expanded and widened. In addition, starting at the *mid-blastula stage* (i.e., at about 9 hpf), the embryos began to rotate within the fertilization envelope, which indicated the presence of cilia on the cell surfaces (for details on cilia see the ciliogenesis section below).

By 11 hpf, the fertilization envelope surrounding the embryos started to rupture (Figure 5D). This indicated that the embryos reached the *hatched blastula stage*. Rupture of the fertilization envelope is ensured by a specific enzyme, called the hatching enzyme, which is synthesized by the animal cells to free the embryos from the fertilization envelope (Lepage et al., 1992). As such, by 12 hpf, all embryos were swimming freely in the cultures. This corresponded to the *swimming blastula stage* (Figure 5E). At this stage, although the embryos remained spherical, the animal-vegetal axis became once again morphologically discernable. The cells at the vegetal pole were now thicker and flatter compared to the rest of the embryo (Figure 5E). This thicker and flatter area corresponds to a specific embryonic structure called the vegetal plate (Ruffins and Ettensohn, 1996). At 13 hpf, the embryos were at the *late swimming blastula stage*. They had elongated along the animal-vegetal axis and their vegetal cells were even thicker, especially on each side of the vegetal pole, resulting in a vegetal plate with a characteristic “V” shape (Figure 5F). Of note, at this stage, the vegetal plate was composed, in a concentric manner, by the small micromeres at the center, followed by the descendants of the large micromeres, and the descendants of the veg2 cells.

#### Gastrulation stages

**FIGURE 6:**
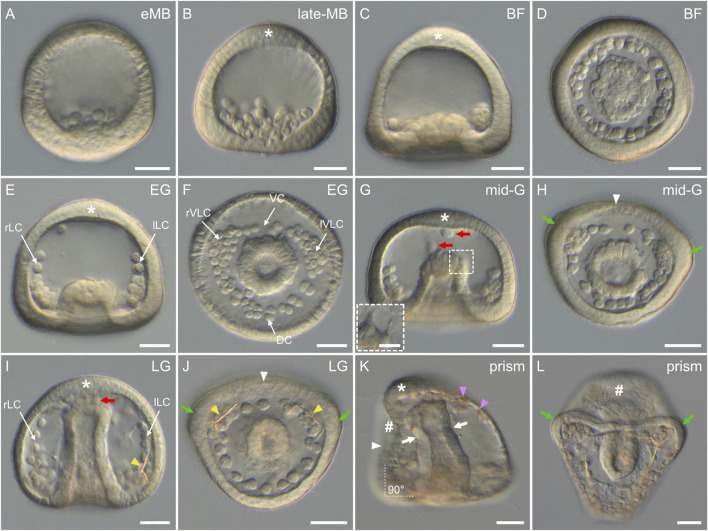
Gastrulation stages of *Paracentrotus lividus* under light microscopy. Developmental stages are as follows: **(A)** early mesenchyme blastula stage (eMB); **(B)** late mesenchyme blastula stage (late-MB); **(C,D)** blastopore formation stage (BF); **(E,F)** early gastrula stage (EG); **(G,H)** mid-gastrula stage (mid-G); **(I,J)** late gastrula stage (LG); **(K,L)** prism stage (prism). In **(A–C,E,G,I,K)**, the embryos are in lateral view with the animal pole up, and in **(K)** the ventral side is left. In **(D,F,H,J,L)**, the embryos are in vegetal view with the ventral side up. In **(B,C,E,G,I,K)**, the asterisk marks the animal (or apical) pole domain. In **(G,I)**, red arrows indicate non-skeletogenic mesoderm cells migrating within the blastocoel. (**(G)** inset) Close-up of the tip of the archenteron of the same embryo as in **(G)**, but at a different focal plane to illustrate non-skeletogenic mesoderm cell ingression. In **(H,J,K)**, the white arrowhead marks the flattening of the ventral ectoderm. In **(H,J,L)**, green arrows highlight the thickened epithelium at the boundary between the vegetal ventral and the vegetal dorsal ectoderm. In **(I,J)**, yellow arrowheads highlight the presence of skeletal elements. In **(K)**, pink arrowheads indicate red-pigmented cells inserted in the aboral ectoderm, and white arrows mark the constriction of the archenteron segregating the esophagus from the stomach. The white dotted lines with the annotation “90°” further indicate the right angle between the ventral and the vegetal ectoderm. In **(K,L)**, the sign “#” highlights the position of the stomodeum, and thus where the mouth will form. Scale bar: **(A–L)** 30 μm; (**(G)** inset) 10 µm. DC: dorsal chain; lLC: left lateral chain; lVLC: left ventrolateral cluster; rLC: right lateral chain; rVLC: right ventrolateral cluster; VC: ventral chain.

In *P. lividus*, as in any other echinoids with micromeres (also called the euechinoids), gastrulation started by the ingression, into the blastocoel, of the descendants of the large micromeres (Figure 6A). This ingression takes place by an epithelial-to-mesenchymal transition and gives rise to the skeletogenic mesoderm (SM) cells (also referred to as the primary mesenchyme cells or PMCs) (Fink and McClay, 1985). Ingression of the SM cells began at the *early mesenchyme blastula stage* (i.e., at around 14 hpf) (Figure 6A), and proceeded until the *late mesenchyme blastula stage* (i.e., at around 17 hpf) (Figure 6B). During this period, the embryos were characterized by an increasing number of SM cells within the blastocoel (up to 32 cells), which, for the time being, remained in the vicinity of the vegetal plate (Figures 6A,B). During this period, the embryos further continued to exhibit, at the vegetal pole, a flattened vegetal plate (Figures 6A,B), which by 17 hpf was only composed of the small micromeres at the center, surrounded by the descendants of the veg2 cells (Ruffins and Ettensohn, 1996; Lyons et al., 2012). By 17 hpf, the descendants of the veg2 cells were also segregated into two different cell tiers, with two distinct cell fates: an inner cell tier surrounding the small micromeres that will develop into non-skeletogenic mesoderm (NSM) cells (also referred to as the secondary mesenchyme cells or SMCs) and an outer cell tier located at the periphery of the vegetal plate that will develop into endoderm cells (Ruffins and Ettensohn, 1996; Lhomond et al., 2012). In addition, at 17 hpf (i.e., at the *late mesenchyme blastula stage*), the embryos were also characterized by the presence, at the animal pole, of a patch of elongated cells (Figure 6B), which will be visible until the end of gastrulation (Figure 6). These cells delimited the animal (or apical) pole domain, within which the neuroectodermal territory will form (Angerer et al., 2011).

The second main morphogenetic movement observed during *P. lividus* gastrulation was subsequently the invagination of the archenteron (i.e., the primordium of the digestive tract). Invagination of the archenteron started at around 18 hpf, at the *blastopore formation stage* (Figures 6C,D). At this stage, the embryos were characterized by the inward bending of the remaining vegetal plate, which created the first opening of the future digestive tract, the blastopore, which will subsequently develop into the anus. This morphogenetic movement has been proposed to be driven by various mechanisms, such as swelling, cell shape changes, pulling, and/or apical secretion, but the exact mechanism at play still remains enigmatic (Ettensohn, 2020). Within the blastocoel, the SM cells concomitantly started migrating, extending filopodia, and attaching and detaching them from the blastocoel wall. By doing so, they progressively adopted a characteristic pattern, forming a ring around the anlage of the archenteron (Figure 6D). At the *early gastrula stage* (20 hpf), the embryos were characterized by an archenteron that had extended within the blastocoel, reaching the approximate level of a quarter of the blastocoel (Figure 6E). This extension was likely due to the invagination of additional NSM cells within the blastocoel. Meanwhile, some SM cells migrated along the inside of the blastocoel wall, towards the animal pole, thereby constituting two lateral chains, the right and the left lateral chains (Figure 6E). In addition, and although the embryos were still relatively spherical at this stage, the ring formed by the SM cells, around the anlage of the archenteron, started to display an asymmetric shape, providing the first morphological landmark for the dorsal-ventral axis. This ring of SM cells was organized into a short ventral and a long dorsal chain, linked by a right and a left aggregate (Figure 6F), which are respectively referred to as the right and the left ventrolateral cluster (Peterson and McClay, 2003).

At the *mid-gastrula stage* (22 hpf), the embryos were characterized by an archenteron length that reached the approximate level of half of the blastocoel (Figure 6G). The archenteron was now likely composed of all of the NSM cells and most of the endoderm cells. Some of the NSM cells, at the tip of the archenteron, started to ingress within the blastocoel (Figure 6G). These cells were undergoing an epithelial-to-mesenchymal transition, extending and projecting thin filopodia to detach from the archenteron (Figure 6G inset). Within the blastocoel, the SM cells still displayed the same characteristic organization as before: an asymmetric ring around the archenteron and two lateral chains along the blastocoel wall. At this stage, the epithelium constituting the ventral side of the embryo further started to flatten, and the epithelium at the boundary between the vegetal ventral and the vegetal dorsal ectoderm started to thicken (Figure 6H), marking the future position of the two first larval arms. At the *late gastrula stage* (24 hpf), the archenteron extended all the way through the blastocoel, up to the blastocoel roof (Figure 6I). At the tip of the archenteron, more delaminating NSM cells were distinguishable (Figure 6I). Within the blastocoel, the SM cells still displayed the same organization as before, and, at the level of the ventrolateral clusters, two skeletal pieces became discernable (Figures 6I,J) (for details on skeleton development see the skeletogenesis section below). At the level of the epithelium, the same features as before were observed, including a flattened ventral ectoderm, thickened areas between the vegetal dorsal and the vegetal ventral ectoderm, and elongated cells constituting the animal pole domain (Figures 6I,J).

In sea urchins, the process of gastrulation ends with the *prism stage* (Kominami and Takata, 2004), which also marks the end of the embryonic period. In *P. lividus*, this stage was observed at around 32 hpf (Figures 6K,L). It was characterized by a notable change in the overall shape of the embryo, as seen in other sea urchin species. The embryo was characterized by a typical triangular (“prism”) shape, with a rounded, elongated dorsal ectoderm and a flattened ventral ectoderm (Figure 6K). The flat ventral ectoderm further formed an almost perfect right angle with the flat vegetal ectoderm (Figure 6K) and contained a small depression right below the apical pole domain (Figures 6K,L). This depression, called the stomodeum, corresponds to the site where the future larval mouth will form (Bergeron et al., 2011). The dorsal ectoderm also contained red-pigmented cells (Figure 6K), which correspond to differentiated NSM cells that are part of the immune system of the embryo and the future larva (Hibino et al., 2006). Within the blastocoel, the archenteron was also bent toward the stomodeum, and a constriction was distinguishable below its tip (Figure 6K). This constriction marked the position of the future cardiac sphincter, which will eventually separate the larval esophagus (above the constriction, close to the stomodeum) from the future larval stomach (below the constriction) (Annunziata et al., 2014).

### Larval development

**FIGURE 7:**
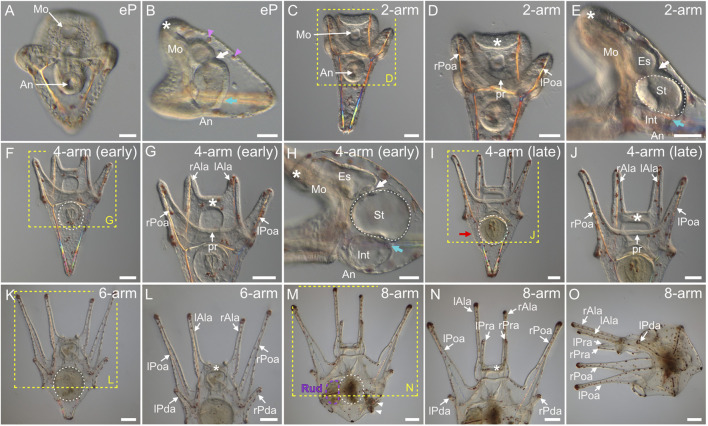
*Paracentrotus lividus* larval development under light microscopy. Developmental stages are as follows: **(A,B)** early pluteus stage (eP); **(C–E)** 2-arm pluteus stage (2-arm); **(F–J)** 4-arm pluteus stage (4-arm); **(K,L)** 6-arm pluteus stage (6-arm); **(M–O)** 8-arm pluteus stage (8-arm). The use of (early) and (late) associated with the stage names simply highlights here more specific periods during the 4-arm pluteus stage. In **(A,C,D,F,G,I–N)**, the larvae are in anterior view with the ventral side up. In **(B,E,H,O)**, the larvae are in left view, with the ventral side left and the anterior side up. In **(B,D,E,G,H,J,L,N)**, the asterisk marks the oral hood located above the mouth. In **(B,E,H)**, the white arrow indicates the cardiac sphincter separating the esophagus and the stomach, and the cyan arrow marks the pyloric sphincter separating the stomach and the intestine. In **(B)**, pink arrowheads highlight red-pigmented cells present within the dorsal ectoderm. In **(D,G,J,L,N)**, the images correspond to close-ups of the regions outlined by yellow boxes in **(C,F,I,K,M)**, respectively. In **(E,F,H,I,K,M)**, the white dotted line outlines the stomach. In **(I)**, the red arrow designates the larval stomach region. In **(M)**, the purple dotted line highlights the adult rudiment, and white arrowheads mark the pedicellariae. Scale bar: **(A–E,H)** 30 μm; **(F,G,I,J)** 50 μm; **(K–O)** 100 µm. An: anus; Es: esophagus; Int: intestine; lAla: left anterolateral arm; lPda: left posterodorsal arm; lPoa: left postoral arm; lPra: left preoral arm; Mo: mouth; pr: postoral region; rAla: right anterolateral arm; rPda: right posterodorsal arm; rPoa: right postoral arm; rPra: right preoral arm; Rud: adult rudiment; St: stomach.

As in many other animals with indirect development, larval development in echinoids begins when the mouth opens (Parichy et al., 2009; Rahman et al., 2012; Carvalho et al., 2021). In *P. lividus*, this event took place at the so-called *early pluteus stage* (i.e., at around 40 hpf), upon the fusion of the tip of the archenteron with the ventral ectoderm, at the level of the stomodeum (Figures 7A,B). At this stage, the archenteron was characterized by a tripartite organization, constituting a functional digestive tract. This tripartite organization was noticeable thanks to the cardiac sphincter, which became clearly apparent at the *early pluteus stage*, as well as to the appearance of a second sphincter, the pyloric sphincter (Figure 7B), which will eventually separate the larval stomach (above the pyloric sphincter) from the larval intestine (below the pyloric sphincter, close to the anus) (Annunziata et al., 2014). At the *early pluteus stage*, the larva also still exhibited red-pigmented cells scattered within the dorsal ectoderm (Figures 7A,B), and these pigmented cells will persist until metamorphosis (Figures 7N,O). Likewise, by this stage, the animal pole domain had extended further into an oral hood (Figure 7B), which protruded above the mouth, and this structure will also persist until metamorphosis (Figure 7N).

By 48 hpf (or 2 days post-fertilization, 2 dpf), the larva had elongated along the dorsal-ventral axis (i.e., from the mouth to the apex) (Figure 7C). The larva had 2 arms positioned posteriorly, on the ventral side, indicating that it reached the *2-arm pluteus stage* (Figures 7C,D). The 2 arms are called the left and the right postoral arms (Thet et al., 2004; Smith et al., 2008), and they delimited, between them, what we named the postoral region. At this stage, the cardiac and pyloric sphincters were clearly constricted, demarcating three compartments within the digestive tract, from the mouth to the anus: the esophagus, the stomach, and the intestine (Figure 7E). At 3 dpf, the larva had grown one additional pair of arms, located anteriorly on the ventral side, at the edges of the oral hood (Figures 7F,G). The emergence of these two additional arms, called the left and the right anterolateral arms (Thet et al., 2004; Smith et al., 2008), marked the beginning of the *4-arm pluteus stage*. At this stage, the epithelium of the digestive tract became thinner (Figure 7H), and the sphincters began to function (for details on muscle development see the myogenesis section below). Starting at the *4-arm pluteus stage* the larvae thus began to feed, as demonstrated by the presence of algae in their stomach and intestine, while in younger larvae, algae were present exclusively in the mouth but were not swallowed (data not shown).

Between 3 and 10 dpf, no significant morphological changes were detected, and the larvae thus remained at a so-called *4-arm pluteus stage* until 10 dpf (Figures 7I,J). However, between 3 and 10 dpf, the four arms of the larvae elongated (Figures 7G,J) and their stomach increased in size (Figures 7F,I), thereby leading to the broadening of the larval apex, at the level of the stomach, along both the anterior-posterior and the left-right axes (Figure 7I). As development proceeded, the only part of the digestive tract that continued to enlarge until metamorphosis was the stomach and its surrounding larval ectodermal region (Figures 7I–O). The development of the digestive tract will thus not be discussed any further. After the *4-arm pluteus stage*, the larvae developed a third pair of arms called the left and the right posterodorsal arms (Thet et al., 2004; Smith et al., 2008). These arms formed in a posterior and dorsal position relative to the anterolateral arms, and their emergence marked the beginning of the *6-arm pluteus stage* (Figures 7K,L), which began at around 10 dpf and lasted until approximately 15 dpf. Subsequently, starting at around 15 dpf, the larva developed a fourth and last pair of arms, called the left and the right preoral arms (Thet et al., 2004; Smith et al., 2008). The preoral arms developed posterior to the anterolateral arms, on the ventral side of the oral hood, and they will be maintained, along with the other three pairs of arms, until metamorphosis. The development of the preoral arms indicated that the larvae entered the *8-arm pluteus stage*, which lasted for about 2 weeks (Figures 7M–O). Through the *6-arm* and *8-arm pluteus stages*, a structure on the left side of the stomach, called the rudiment and corresponding to the anlage of the future sea urchin adult, further considerably increased in size (Figure 7M) (for details on rudiment formation see the coelomogenesis and adult rudiment ontogeny sections below). Due to the presence of this structure and its growth through time, the stomach region of the *8-arm pluteus stage* larva expanded and acquired a cuboidal shape (Figures 7M,O). In addition to the rudiment, the larva further developed additional structures of the future adult on its right side, such as the pedicellariae and the genital plates (for details on the formation of other adult structures see the development of complementary adult structures section below).

### Ciliogenesis in *Paracentrotus lividus* embryos and larvae

**FIGURE 8:**
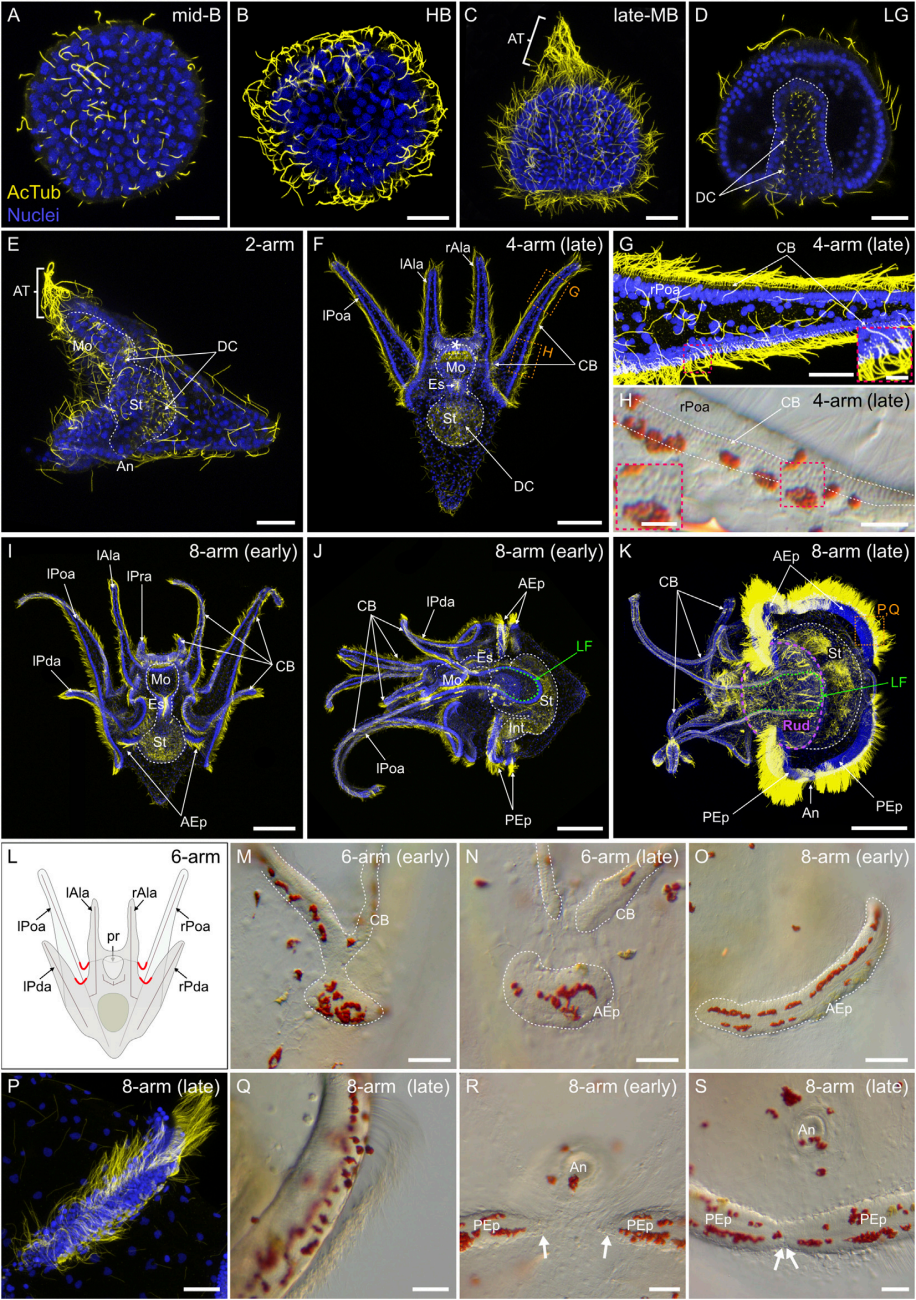
Ciliogenesis in *Paracentrotus lividus* during the embryonic and larval periods. Developmental stages are as follows: **(A)** mid-blastula stage (mid-B); **(B)** hatched blastula stage (HB); **(C)** late mesenchyme blastula stage (late-MB); **(D)** late gastrula stage (LG); **(E)** 2-arm pluteus stage (2-arm); **(F–H)** 4-arm pluteus stage (4-arm); **(I–K, O–S)** 8-arm pluteus stage (8-arm); **(L–N)** 6-arm pluteus stage (6-arm). The use of (early) or (late) associated with the stage names simply highlights here more specific periods during the 4-, 6- or 8-arm pluteus stages. In **(A–G,I–K,P)**, images are maximum intensity projections of confocal z-stacks of embryos and larvae co-labeled for acetylated α-tubulin (cilia; yellow) and DNA (nuclei; blue), and they correspond to projections of the entire specimen, except for **(D)** that is a cross-section through the embryo. In **(H,M–O,Q–S)**, images were acquired using light microscopy. **(L)** Schematics of a larva at the 6-arm pluteus stage illustrating in red the areas of the ciliary band that will bud to form the epaulettes. In **(A–D)**, embryos are in lateral view with the animal pole up. In **(E,J,K)**, larvae are in left view, with the anterior side up and the ventral side left. In **(F,I)**, larvae are in anterior view, with the ventral side up. **(G,H)** Close-ups of the ciliary band of a late 4-arm pluteus stage larva, corresponding to the regions outlined by orange boxes in **(F)**. (**(G)** inset, **(H)** inset**)**, Close-ups of the ciliary band to highlight the distribution of cuboidal cells and their associated cilia. **(M–O)** Close-ups of a ciliary band bud and its related epaulette during the 6- and 8-arm pluteus stages. **(P,Q)** Close-ups of the anterior epaulette in a larva at a late 8-arm pluteus stage, corresponding to the region outlined by the orange box in **(K)**. **(R,S)** Close-ups of the posterior epaulettes in a larva at the begin and at the end of the 8-arm pluteus stage, respectively. In **(D)**, the white dotted line outlines the archenteron and, in **(E) (F)**, **(I–K)**, the digestive tract. In **(F)**, the white asterisk marks the oral hood. In **(H)**, white dotted lines delineate the three rows of cuboidal cells and their associated cilia. In **(J,K)**, the green dotted line indicates the position of the lateral field. In **(K)**, the purple dotted line outlines the adult rudiment. In **(M–O)**, the white dotted line highlights the ciliary band and the developing epaulette. In **(R)**, white arrows mark the posterior end of the two posterior epaulettes and in **(S)** the site of fusion of the two posterior epaulettes. Scale bar: **(A–E,G,M–N,P–S)** 30 μm; **(F)** 50 μm; (**(G)** inset, **(H)** inset) 7.5 µm; **(H)** 15 μm; **(I–K)** 150 µm. AcTub: acetylated α-tubulin; AEp: anterior epaulette; An: anus; AT: apical tuft; CB: ciliary band; DC: digestive tract cilia; Es: esophagus; Int: intestine; lAla: left anterolateral arm; LF: lateral field; lPda: left posterodorsal arm; lPoa: left postoral arm; lPra: left preoral arm; Mo: mouth; PEp: posterior epaulette; pr: postoral region; rAla: right anterolateral arm; rPda: right posterodorsal arm; rPoa: right postoral arm; Rud: adult rudiment; St: stomach.

Upon hatching, sea urchin embryos and larvae swim in the water column using ciliary beating (Strathmann, 1971). To describe the ciliated structures of *P. lividus* embryos and larvae, we next performed an immunohistochemical assay using anti-acetylated α-tubulin antibodies. During embryonic development, *P. lividus* embryos first started to develop cilia at the *mid-blastula stage* (Figure 8A). It was only at this stage that we started detecting, on the apical surface of some embryonic cells, the presence of a unique cilium (Figure 8A). At this stage, already, these cilia were further functional and capable of metachronal beating, as demonstrated by the rotative movement at the *mid-blastula stage* of the embryos within their fertilization envelope (data not shown). As development proceeded, every cell of the embryo eventually formed a unique cilium (Figure 8B), and, upon hatching, these cilia allowed the embryos to swim freely in the water column. Furthermore, starting at the *early mesenchyme blastula stage*, the cilia located in the animal pole domain grew longer than those present on other cells of the embryo. By the *late mesenchyme blastula stage*, these animal pole cilia reached a size of about 100 µm and formed a conspicuous patch of long, immotile cilia referred to as the apical tuft (Figure 8C). During gastrulation, the cells invaginating into the blastocoel and constituting the archenteron further conserved their short cilia on their apical surface. These short cilia thus lined the lumen of the archenteron (Figure 8D) and subsequently that of the functional digestive tract of the larva, until metamorphosis (Figures 8E,F,I–K).

At the *2-arm pluteus stage*, the larvae still exhibited the long immotile cilia of the apical tuft and cilia on all other cells of the outer epithelium as well as in the lumen of the archenteron (Figure 8E). As development proceeded, the long immotile cilia of the apical tuft regressed and completely disappeared during the *4-arm pluteus stage* (Figure 8F). In addition, the distribution of the cilia on the outer epithelium of the larva progressively changed. During the *4-arm pluteus stage*, the epidermal cells constituting the ventral and dorsal epithelium remained squamous and shortened their cilia, while those along the lining of the anterolateral and postoral arms as well as those on the ventral edge of the oral hood (i.e., all the cells located at the interface between the ventral and the dorsal ectoderm) adopted a cuboidal shape and continued to bear medium-sized cilia (Figure 8F). Furthermore, the cuboidal cells started to organize themselves into a compact row (Figures 8G,H), resulting in a significant local increase of ciliary density (Figures 8F,G). This row of tightly-packed ciliated cells is commonly referred to as the ciliary band (Slota et al., 2020), although some authors refer to it as the neotroch (Nielsen, 1998).

During the *6-arm* and *8-arm pluteus stages*, the ciliary band extended along the newly developing posterodorsal and preoral pairs of arms (Figures 8I–K), and additional ciliated structures, called the epaulettes, emerged from the ciliary band (Figures 8I–S). At the *6-arm pluteus stage*, patches of ciliary band spread towards the dorsal side of the larva, on both the right and the left sides (Figure 8L). These patches spread from the hinges between the anterolateral and the posterodorsal arms as well as between the postoral arms and the postoral region (Figures 8L,M). As development proceeded, the emerging patches progressively isolated themselves from the ciliary band (Figure 8N) and elongated along the left-right axis of the larva (Figure 8O). They further migrated either anteriorly, spreading from underneath the anterolateral and the posterodorsal arms, or posteriorly, spreading from below the postoral arms and the postoral region. Thus, starting early during the *8-arm pluteus stage*, the larva was characterized by a complex ciliary band outlining the oral region and by four large, independent patches of ciliated cells corresponding to the two anterior and the two posterior epaulettes (Figures 8I,J,O). During the *8-arm pluteus stage*, the four epaulettes further continued to extend laterally, adopting an arc-shaped conformation (Figures 8J,K). They also thickened considerably and increased their ciliary density (Figures 8J,K,P,Q). During this process, the cilia of the epaulettes grew in length, reaching a size longer than the cilia of the ciliary band (Figure 8K). The two posterior and the two anterior epaulettes also eventually fused, respectively, at the level of the larval anus (Figures 8R,S) and on the opposite anterior side (Figure 8K). Late during the *8-arm pluteus stage*, the epaulettes thus covered most of the circumference of the larval stomach region (Figure 8K), being interrupted only in two areas on each side of the larva. These two areas are referred to as the lateral fields (Gosselin and Jangoux, 1998), and they were outlined by two large folds of ciliary band located on each side of the larva between the postoral and the posterodorsal arms (Figures 8J,K).

### Skeletogenesis in *Paracentrotus lividus* embryos and larvae

**FIGURE 9:**
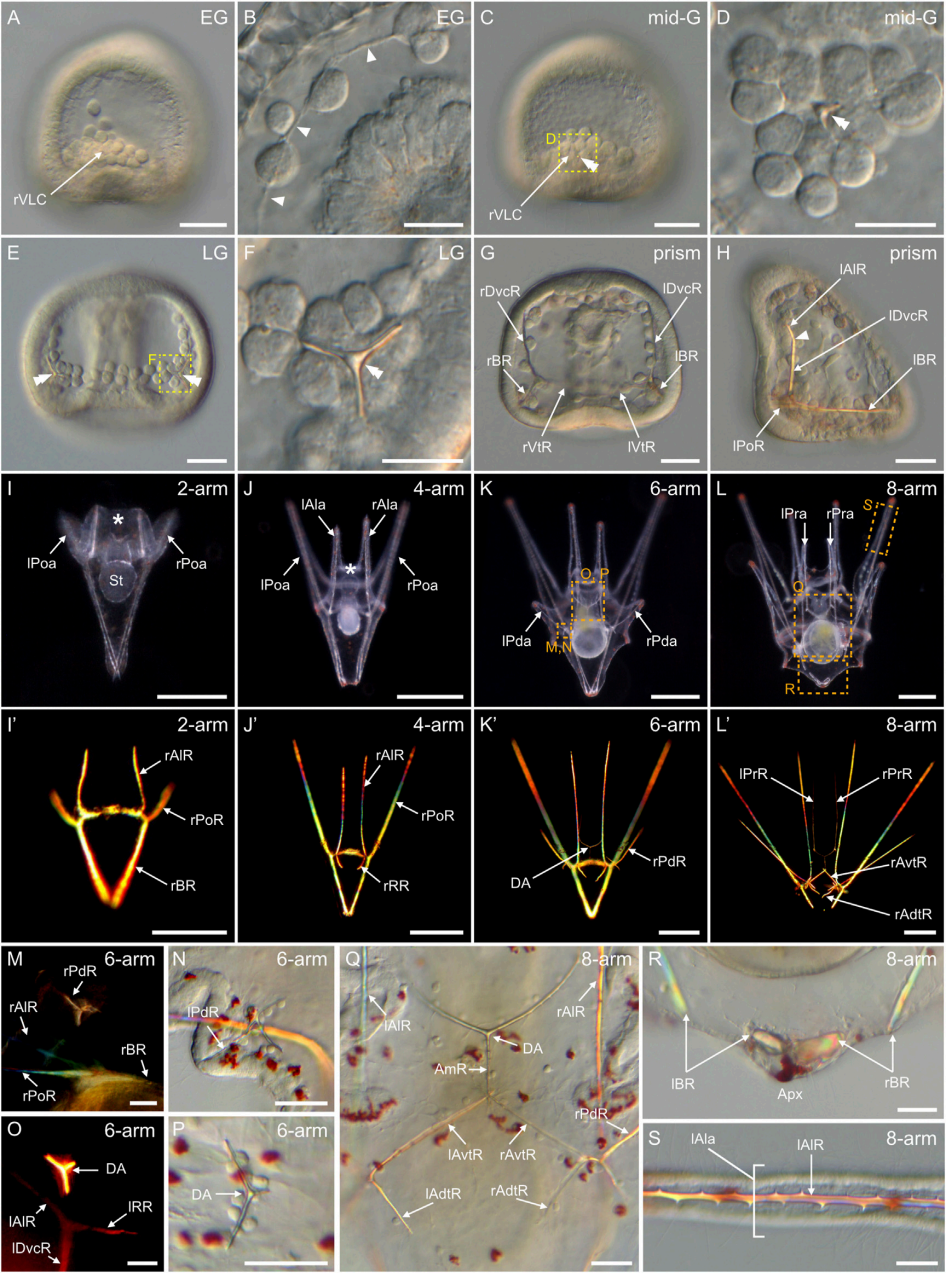
Skeletogenesis in *Paracentrotus lividus* during the embryonic and larval periods. Developmental stages are as follows: **(A,B)** early gastrula stage (EG); **(C,D)** mid-gastrula stage (mid-G); **(E,F)** late gastrula stage (LG); **(G,H)** prism stage (prism); **(I,I')** 2-arm pluteus stage (2-arm); **(J,J′)** 4-arm pluteus stage (4-arm); **(K,K',M–P)** 6-arm pluteus stage (6-arm); **(L,L',Q–S)** 8-arm pluteus stage (8-arm). In **(A–L,N,P–S)**, images were acquired using light microscopy either in bright-field for **(A–H,N,P–S)**, or dark-field for **(I–L)**. In **(I'–L',M,O)**, images were obtained using polarized light to highlight the skeletal elements. In **(A,C,H)**, embryos are in right view, with the animal pole up and, for **(H)**, with the ventral side left. In **(E,G)**, embryos are in ventral view, with the animal pole up. In **(I–L′)**, larvae are in anterior view, with the ventral side up. **(B)** Close-up of the skeletogenic mesoderm cells and their related filopodia in a ventral chain. **(D,F)** Close-ups of a ventrolateral cluster to highlight the rhombohedral crystal in **(D)** and the triradiate spicule in **(F)**, the two images corresponding, respectively, to the regions highlighted by yellow boxes in **(C)** and **(E)**. **(M–P)** Close-ups of the regions outlined by orange boxes in **(K)** with larvae in lateral view, except for (M) where the larva is in anterior view. **(M,N)** Close-ups of the developing posterodorsal spicule. **(O,P)** Close-ups of the developing dorsal arch. **(Q–S)** Close-ups of the regions highlighted by orange boxes in **(L)** with larvae in anterior view. **(Q)** Close-up of the skeletal elements located in the vicinity of the larval digestive tract. **(R)** Close-up of the body rods in the most dorsal region of the larva. **(S)** Close-up of the non-fenestrated spicule of an anterolateral arm. In **(B)**, arrowheads highlight the filopodia extended by skeletogenic mesoderm cells. In **(C–F)**, double arrowheads mark the skeletal elements forming in the ventrolateral clusters. In **(H)**, the arrowhead highlights the limit between the right dorsoventral connecting rod and the right anterolateral rod. In **(I,J)**, the asterisk marks the oral hood. Scale bar: **(A,C,E,G,H,M–S)** 30 μm; **(B,D,F)** 15 μm; **(I)** 100 μm; **(J–L,J′–L′)** 200 µm. AmR: anteromedial rod; Apx: apex; DA: dorsal arch; lAdtR: left anterodorsal transverse rod; lAla: left anterolateral arm; lAlR: left anterolateral rod; lAvtR: left anteroventral transverse rod; lBR: left body rod; lDvcR: left dorsoventral connecting rod; lPoa: left postoral arm; lPoR: left postoral rod; lPda: left posterodorsal arm; lPdR: left posterodorsal rod; lPra: left preoral arm; lPrR: left preoral rod; lRR: left recurrent rod; lVtR: left ventral transverse rod; rAdtR: right anterodorsal transverse rod; rAla: right anterolateral arm; rAlR: right anterolateral rod; rAvtR: right anteroventral transverse rod; rBR: right body rod; rDvcR: right dorsoventral connecting rod; rPoa: right postoral arm; rPoR: right postoral rod; rPda: right posterodorsal arm; rPdR: right posterodorsal rod; rPra: right preoral arm; rPrR: right preoral rod; rRR: right recurrent rod; rVLC: right ventrolateral cluster; rVtR: right ventral transverse rod; St: stomach.

In *P. lividus*, the formation of the larval endoskeleton began at the *early gastrula stage*, with the SM cells extending filopodia towards each other (Figures 9A,B). The extension of filopodia was detectable initially in the ventrolateral clusters and in the ventral and dorsal chains (Figure 9B), before it took place also in the lateral chains. The filopodia fused the SM cells together and generated long syncytial cables, onto which a spicular matrix subsequently accumulated, allowing calcium carbonate deposition and formation of calcified skeletal elements (Figures 9C–L') (Okazaki, 1965; Decker and Lennarz, 1988). The first of these calcified elements appeared at the *mid-gastrula stage* in the two ventrolateral clusters, in the form of a calcite crystal of rhombohedral shape (Figures 9C,D). By the *late gastrula stage*, each crystal had developed three smooth, rounded rods, forming a triradiate spicule on each side of the embryo (Figures 9E,F). Each rod had elongated in the direction of the a-axis of the calcite crystal (Kitajima and Urakami, 2000) and formed an angle of 120° with the two other rods. By the *prism stage*, each rod had further elongated along the a-axes, on both the left and right sides of the embryo, generating two mirror triradiate skeletal pieces (Figures 9G,H). Each of these pieces was thus constituted of three elongated skeletal rods referred to as: 1) the ventral transverse rod, which grew along the vegetal and ventral side of the embryo (Figure 9G), 2) the dorsoventral connecting rod, which elongated towards the animal pole and along the ventral side of the embryo (Figures 9G,H), and 3) the body rod, which formed along the vegetal side of the embryo towards the apex (Figures 9G,H) (MacBride, 1911; Smith et al., 2008). Of note, at this stage, we further observed that the dorsoventral connecting rods were bent towards the ventral ectoderm at the level of the stomodeum (Figure 9H). The site of this bent marked the limit between the dorsoventral connecting rods and the anlage of the anterolateral rods (Figure 9H), which will later support the anterolateral arms. We further observed, at this stage, the anlage of the postoral rods, which will support the postoral arms. The postoral rods were pointing towards the ventral side of the embryo, in extension of the body rods (Figure 9H).

During the subsequent larval period, the organization of the endoskeleton became increasingly more complex (Figures 9I–L'). At the *2-arm pluteus stage*, no additional skeletal pieces were observed, but each of the existing rods elongated to varying degrees. For instance, the postoral rods extended to form the anlage of the postoral arms, and the body rods elongated to touch each other at their dorsal extremities, giving the larval apex its distinctive pointy shape (Figures 9I,I'). Likewise, the anterolateral rods expanded to support the oral hood (Figure 9I), however they only individualized and spread into the anterolateral arms at the *4-arm pluteus stage*. The *4-arm pluteus stage* was further marked by a significant extension of the postoral arms and rods (Figures 9J,J') as well as by the emergence of another branching at the junction between the dorsoventral connecting rods and the anterolateral rods. This new branching resulted in two short rods extending towards the apex on each of the lateral sides of the stomach (Figures 9J', O). These two short rods are respectively referred to as the right and the left recurrent rods (MacBride, 1911).

Thereafter, three new skeletal elements appeared, each one of them independent of the rods already in place. These new skeletal elements were the two posterodorsal spicules and the dorsal arch (MacBride, 1911; Smith et al., 2008). The posterodorsal spicules formed on each side of the larva, just above the junctions between the ventral transverse, dorsoventral connecting, postoral, and body rods (Figures 9K,K',M,N). The dorsal arch formed just above the stomach, in a dorsal-anterior position relative to the esophagus (Figures 9K,K',O,P). Development of these new skeletal elements followed the same developmental trajectory as the previous skeletal rods. Between the *4-arm* and *6-arm pluteus stages*, they emerged as three new rhombohedral-shaped crystals, from which three smooth, rounded rods developed, one along each of the three a-axes (Figures 9M,O). At the level of the posterodorsal spicules, the rod facing out from the larva and referred to as the posterodorsal rod (Smith et al., 2008), will later support the posterodorsal arm (Figures 9K,K',M,N). This rod extended earlier than the other two rods of the posterodorsal spicules, and its extension marked the beginning of the *6-arm pluteus stage* (Figure 9K'). At the level of the dorsal arch, the two rods facing the oral hood also extended earlier than the one facing the apex (Figures 9O,P). These two rods extended first laterally towards the anterolateral rods. Then, once they reached the edge of the esophagus, they bent along a 120° angle and extended ventrally, thereby giving rise to the anlage of the preoral rods (Figure 9K').

As development proceeded, the preoral rods protruded outside the ventral side of the oral hood and generated the preoral arms, hence marking the beginning of the *8-arm pluteus stage* (Figures 9L,L'). At this stage, the posterodorsal rods were considerably extended, and additional rods were formed from the posterodorsal spicules and the dorsal arch. At the level of the posterodorsal spicules, for instance, two smooth rods were now facing the inside of the larval body and elongated both above and below the stomach, into what we referred to as the anteroventral and the anterodorsal transverse rods (Figures 9L,L’, Q). Likewise, at the level of the dorsal arch, the smooth rod facing the apex extended above the stomach into a rod that we named the anteromedial rod (Figures 9L,L',Q). The *8-arm pluteus stage* was further marked, at the tip of the apex, by the rupture of the body rods (Figure 9R), which was concomitant with a significant enlargement of the larval body at the level of the stomach region (Figure 9L). It is worth noting that we did not identify any fenestrated skeletal rods in the *P. lividus* larva. Instead, the rods of the *P. lividus* larva were full and spiny (Figure 9S), which is similar to what has been observed in some other sea urchin species (Wray, 1992). Supplementary Figure S2 provides a scheme highlighting all the different larval skeletal rods along with fixed landmarks to facilitate identification.

### Myogenesis in *Paracentrotus lividus* embryos and larvae

**FIGURE 10:**
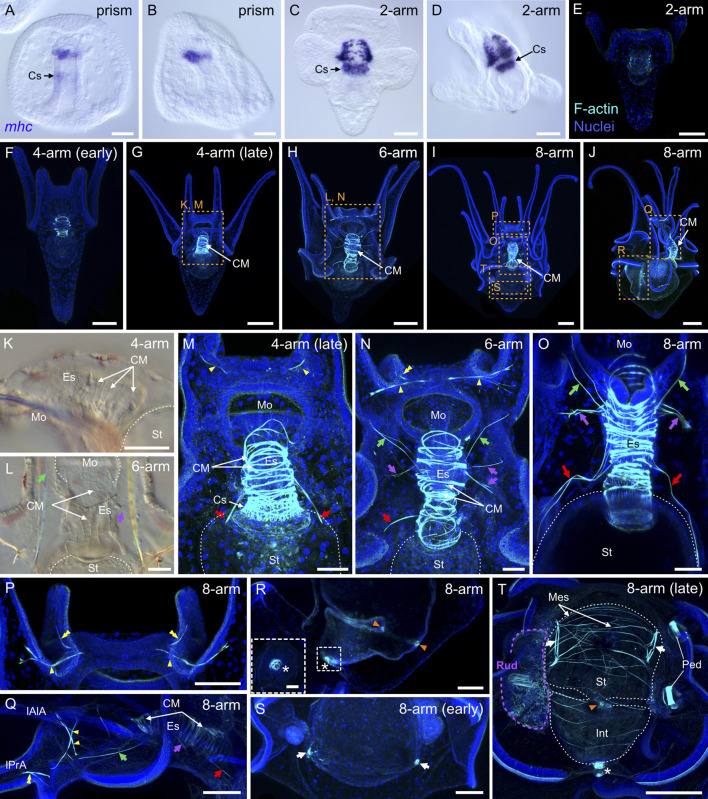
Myogenesis in *Paracentrotus lividus* during the embryonic and larval periods. Developmental stages are as follows: **(A,B)** prism stage (prism); **(C–E)** 2-arm pluteus stage (2-arm); **(F,G,K,M)** 4-arm pluteus stage (4-arm); **(H,L,N)** 6-arm pluteus stage (6-arm); **(I,J,O–T)** 8-arm pluteus stage (8-arm). The use of (early) or (late) associated with the stage names simply highlights here more specific periods during the 4- or 8-arm pluteus stages. In **(A–D)**, images were acquired using bright-field, differential interference contrast light, and they correspond to embryos and larvae labeled for the muscle terminal differentiation gene *myosin heavy chain* (*mhc*). In **(E–J,M–T)**, images are maximum intensity projections of confocal z-stacks of larvae co-labeled for F-actin (muscles; cyan) and DNA (nuclei; blue), and they correspond to projections of the entire specimen. In **(K,L)**, images were acquired using bright-field light microscopy. In **(A)**, the embryo is in ventral view, with the animal pole up. In **(B,D,J,K,Q,R)**, embryos and larvae are in left view, with either the animal pole up and the ventral side left in **(B)** or with the anterior pole up and the ventral side left in **(D,K,Q,R)** or with the anterior pole right and the ventral side up in **(J)**. In **(C,E–I,L–P,S,T)**, larvae are in anterior view, with the ventral side up. **(K–T)** Close-ups of the regions outlined by orange boxes in **(G–J)**. **(K–O,Q)** Close-ups of the esophageal region. **(P)** Close-up of the oral hood. **(R)** Close-up of the intestinal region. (**(R)** inset) Close-up of the anal sphincter. **(S,T)** Close-ups of the stomach region. In **(K–O,T)**, the white dotted line outlines the larval digestive tract. In **(L,N,O,Q)**, green arrows highlight the posterior dilator muscles, and purple arrows mark the lateral muscles. In **(M–O,Q)**, red arrows indicate the ventrolateral processes (or longitudinal musculature). In **(M,N,P,Q)**, yellow arrowheads mark the anterior dilator muscles (or star-shaped muscles). In **(N,P,Q)**, yellow double arrowheads highlight the preoral dilator muscles. In **(R,T)**, orange arrowheads mark the pyloric sphincter, and the white asterisk marks the anal sphincter. In **(S,T)**, white arrows indicate the lateral stomach muscles. In **(T)**, the purple dotted line delineates the adult rudiment. In **(H,J,M,N,P,Q,S,T)**, the F-actin staining detected along the ciliary band and the epaulettes corresponds to a counterstain of the apex of the cuboidal cells and thus not to muscle cells. Scale bar: **(A–D,M–O)** 30 μm; **(E,F,K,L,P–T)** 50 μm; **(G–J)** 100 μm; (**(R)** inset) 10 µm. CM: circumesophageal muscle; Cs: cardiac sphincter; Es: esophagus; Int: intestine; lAlA: left anterolateral arm; lPrA: left preoral arm; Mes: mesentery; Mo: mouth; Ped: pedicellariae; Rud: adult rudiment; St: stomach.

We next investigated muscle development in *P. lividus* during the embryonic and larval periods. For this, we used two complementary approaches. First, we carried out *in situ* hybridization assays for the muscle terminal differentiation gene *myosin heavy chain* (*mhc*) (Wessel et al., 1990). Second, we took advantage of the enrichment of F-actin in muscles and performed F-actin immunohistochemistry assays using a rhodamine-labeled phalloidin derivative. The expression of the *mhc* gene was the first to be detected in embryos, at the *prism stage* (Figures 10A,B). At this stage, *mhc* staining was detected at the tip of the gut on the oral side and, more subtly, at the level of the cardiac sphincter (Figures 10A,B). By the *2-arm pluteus stage*, *mhc* staining was visible in the muscle fibers extending around the esophagus as well as in those surrounding the cardiac sphincter (Figures 10C,D). At this stage, we also started detecting phalloidin staining, which revealed a signal in circumferential fibers surrounding the esophagus (Figure 10E). The signal in these muscle fibers, referred to as the circumesophageal muscles (Annunziata et al., 2014), became more conspicuous right at the beginning of the *4-arm pluteus stage* (Figure 10F), the stage at which the larvae started feeding, swallowing microalgae using the contraction and relaxation movements of the muscles surrounding the esophagus and the cardiac sphincter (Supplementary Video S1). During the *4-arm pluteus stage*, the anatomical organization of the larval muscles did not significantly change (Figures 10F,G,K,M). However, the circumesophageal muscles accumulated more F-actin (Figures 10F,G), increased in number around the esophagus (Figure 10M), and started to be distinguishable as a series of thin stripes under a regular transmitted light microscope (Figure 10K).

By the end of the *4-arm pluteus stage* and during the *6-arm pluteus stage*, several additional muscles appeared. These muscles were located chiefly in the oral hood and along the circumesophageal muscles (Figures 10G,H,L–N), and they continued to develop during the *8-arm pluteus stage* (Figures 10I,J,O–Q). In the oral hood, a first new pair of muscles emerged at the base of the left and the right anterolateral arms (Figure 10M). These muscles were identified as those called the anterior dilator muscles by Strathmann (1971) or the star-shaped muscles by Dyachuk and Odintsova (2013). By the *8-arm pluteus stage*, these muscles were joined by two additional left and right muscles projecting into the left and the right preoral arms (Figures 10N,P,Q). We here referred to these muscles as the preoral dilator muscles, with the idea that they likely have the same function as the anterior dilator muscles. At the level of the circumesophageal muscles, between the end of the *4-arm pluteus stage* and the *8-arm pluteus stage*, three additional pairs of lateral muscles further formed, on both the left and the right sides of the larva (Figures 10L–O,Q). The most ventral of these muscle pairs, i.e., the posterior dilator muscles (Strathmann, 1971), extended to the rim of the mouth and connected the anterior dilator muscles (Figures 10L,N,O,Q). The intermediate pairs, which we referred to as the lateral muscles, projected to the larval epidermis (Figures 10L,N,O,Q). Last, the most dorsal pair, referred to as the ventrolateral processes (Burke and Alvarez, 1988) or the longitudinal musculature (Dyachuk and Odintsova, 2013), extended dorsally and connected the esophagus with the stomach (Figures 10M–O,Q). At the *8-arm pluteus stage*, we were further able to identify additional muscles associated with the digestive tract. Among these muscles were those associated with the pyloric and anal sphincters (Figures 10R,T). These muscles initially formed by the end of the *4-arm pluteus stage* (data not shown), but became clearly distinguishable with phalloidin staining only much later in development. In addition, while the muscles associated with the cardiac sphincter were found embedded within the circumesophageal muscles (Figure 10M), those of the pyloric and anal sphincters corresponded to individual and independent ring-shaped muscles (Figure 10R). At the *8-arm pluteus stage*, we also found two other types of muscles to be associated with the digestive tract: a pair of muscles located on each side of the stomach that we named the lateral stomach muscles (Figures 10S,T) and several thin muscle fibers extending around the digestive tract that we referred to as mesenteries (Figure 10T).

### Coelomogenesis in *Paracentrotus lividus* embryos and larvae and emergence of the adult rudiment

**FIGURE 11:**
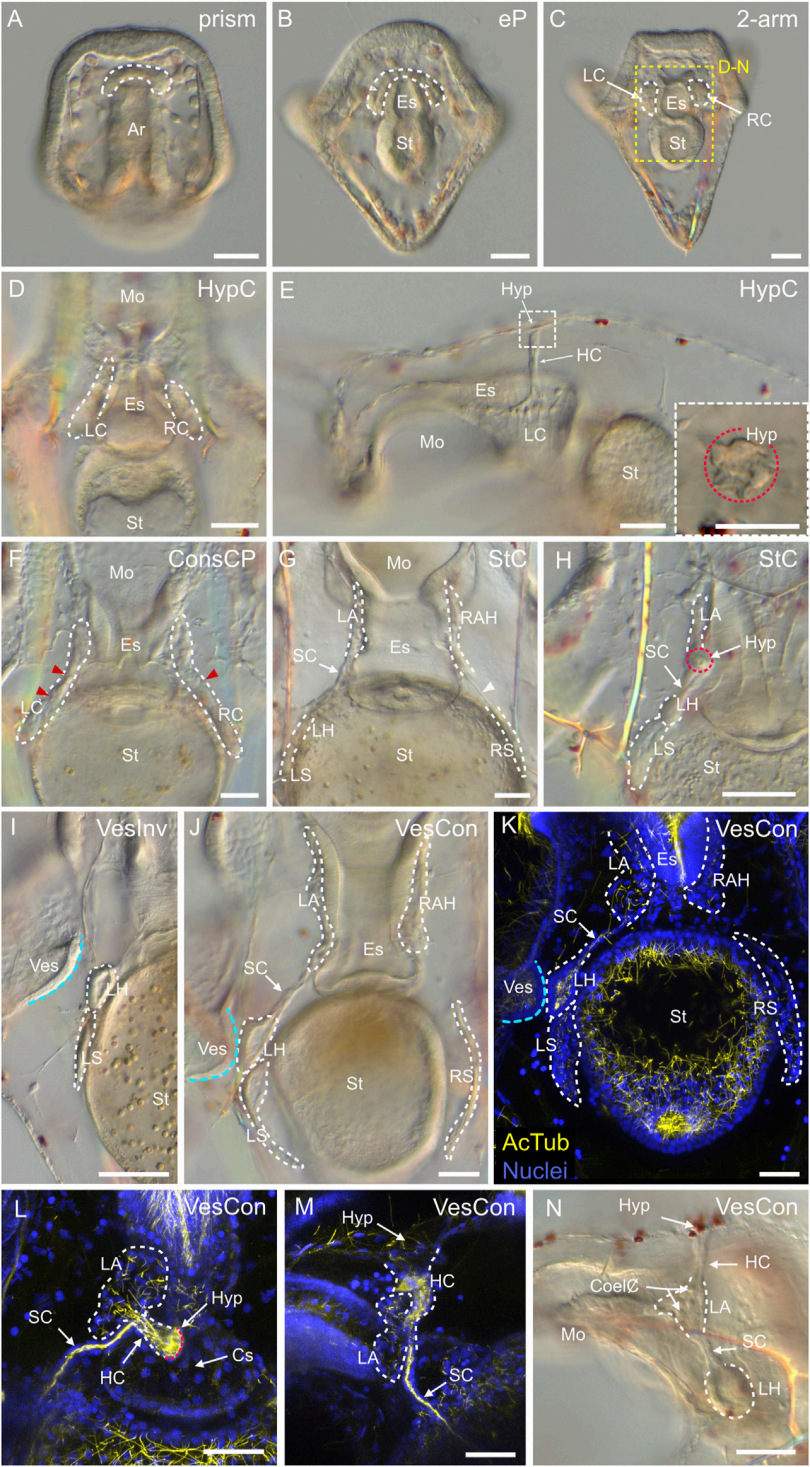
Coelomogenesis in *Paracentrotus lividus* during the embryonic and larval periods. Developmental stages are as follows: **(A)** prism stage (prism); **(B)** early-pluteus stage (eP); **(C)** 2-arm pluteus stage (2-arm); **(D,E)** hydroporic canal stage (HypC); **(F)** constricted coelomic pouch stage (ConsCP); **(G,H)** stone canal stage (StC); **(I)** vestibule invaginated stage (VesInv); **(J–N)** vestibule contact stage (VesCon). In **(A–J,N)**, images were acquired using bright-field light microscopy. In **(K–M)**, images are maximum intensity projections of confocal z-stacks of larvae co-labeled for acetylated α-tubulin (cilia; yellow) and DNA (nuclei; blue), and they correspond to projections of the entire specimen. In **(A)**, the embryo is in ventral view, with the animal pole up. In **(B–D,F–L)**, larvae are in anterior view, with the ventral side up. In (**(E)** inset, **(M,N)**), larvae are in left view, with the ventral side to the left. **(D–N)** Close-ups of the region outlined by the yellow box in **(C)**. **(D,F,G,J,K)** Close-ups of the larval digestive tract to show the right and the left coelomic pouches. **(E,H,I,L–N)** Close-ups of the left coelomic pouch region. In **(A–D,F–N)**, white dotted lines outline the right and the left coelomic pouches as well as their derivatives. In (**(E)**, inset **(H,L)**), the red dotted line delineates the hydropore. (**(E)** inset) Close-up of the hydropore in anterior view. In **(F)**, red arrowheads highlight constrictions within the coelomic pouches. In **(G)**, the white arrowhead marks the isthmus between the right axohydrocoel and the right somatocoel. In **(I–K)**, the cyan dotted line highlights the vestibule. Scale bar: **(A–N)** 30 μm; (**(E)** inset) 7.5 µm. AcTub: acetylated α-tubulin; Ar: archenteron; Coel: coelomocyte; Cs: cardiac sphincter; Es: esophagus; HC: hydroporic canal; Hyp: hydropore; LA: left axocoel; LC: left coelomic pouch; LH: left hydrocoel; LS: left somatocoel; Mo: mouth; RAH: right axohydrocoel; RC: right coelomic pouch; RS: right somatocoel; SC: stone canal; St: stomach; Ves: vestibule.

One important feature of *P. lividus* larval development was the ontogeny of the larval coeloms, from which the adult body plan subsequently arose. As in many other sea urchins, coelomogenesis in *P. lividus* took place by enterocoely, meaning that the coelomic compartments initially formed by evagination from the archenteron (Technau and Scholz, 2003). This process started at the *prism stage*, at the tip of the archenteron (Figure 11A). The evaginating cells thus formed a coelomic sac covering the tip of the archenteron. Then, this sac progressively extended laterally, establishing, by the *early pluteus stage*, a bilobed-shaped structure (Figure 11B). At the *early pluteus stage*, the lobes were still connected to each other by an isthmus, and one lobe was positioned to the right side, while the other one was to the left side, of the digestive tract (Figure 11B). By the *2-arm pluteus stage*, the lobes were completely separated from one another (Figure 11C), leading to the emergence of two independent, epithelial coelomic pouches, one on each side of the digestive tract, and which are commonly referred to as, respectively, the right and the left coelomic pouch (Molina et al., 2013).

At subsequent stages, we noticed a certain heterochrony between the trajectories of the coeloms and the larval development, although we always observed the same sequence of events for both the coeloms and the larval development. We thus decided, for now long, not to refer to the larval stages anymore when describing the development of the coeloms and their subsequent related structures. Instead, we decided to use a specific staging scheme that is included as a subsection in Figure 3. Following the *2-arm pluteus stage*, and despite the heterochrony, we thus always observed at the level of the coeloms: 1) a progressive elongation of the right and the left coelomic pouches along the esophagus (Figure 11D) and 2) the emergence of a duct-like structure extending from the left coelomic pouch towards the anterior larval epidermis (Figure 11E). This duct-like structure is referred to as the hydroporic canal, and it opened in the larval epidermis through a hole called the hydropore (Figure 11E) (Luo and Su, 2012). The presence of this anatomical structure defined what we referred to as the *hydroporic canal stage*, and the emergence of the hydroporic canal and the hydropore constituted the first landmarks of the left-right asymmetry of the larva, with these two structures forming only on the left side. Moreover, they were the first indication of the formation of the adult water vascular system and the first connection of the left coelomic pouch to the external environment (Luo and Su, 2012).

As development proceeded, both the right and the left coelomic pouches elongated towards the larval apex. By the time they reached the anterior part of the stomach, they displayed several distinct constrictions (Figure 11F). The appearance of these constrictions marked the *constricted coelomic pouch stage*. At this stage, the pouches started to segregate into distinct compartments, and the morphological events taking place in the left coelomic pouch became more elaborate than those occurring in the right coelomic pouch. For instance, at the *constricted coelomic pouch stage*, while the left coelomic pouch was characterized by two constrictions, the right coelomic pouch only had one (Figure 11F). At the subsequent stage, which we named the *stone canal stage*, each pouch had further considerably extended towards the mouth and the middle part of the stomach, and each pouch was now separated into several, clearly distinguishable coelomic compartments, on each side of the larval digestive tract (Figures 11G,H). On the left side, for instance, the coelomic pouch had given rise to three compartments, referred to, from the mouth to the stomach, as the left axocoel, the left hydrocoel, and the left somatocoel (Peterson et al., 2000; Smith et al., 2008). On the right side, the coelomic pouch had formed two compartments, called, from the mouth to the stomach, the right axohydrocoel and the right somatocoel (Peterson et al., 2000). On the left side, the left axocoel was further still connected, anteriorly, to the external environment, through the hydroporic canal and the hydropore, and it was connected, posteriorly, to the left hydrocoel by a thin, tubular structure referred to as the stone canal (Figures 11G,H) (Smith et al., 2008). On the right side, by contrast, the right axohydrocoel and somatocoel were not connected to the external environment, but they were still attached to one another by a thin isthmus (Figure 11G), which however disappeared soon thereafter (Figures 11J,K).

During subsequent development, the left and the right coelomic compartments continued to extend along the anterior-posterior axis of the larva (Figures 11I–K). On the left side, at the level of the larval ectoderm, we further started to observe a thickening and invagination of a portion of the epidermis located at the base of the left postoral and the left posterodorsal arms (Figure 11I). This invagination is referred to as the vestibule (Smith et al., 2008; Heyland and Hodin, 2014), and it will greatly contribute to the development of the adult rudiment (for details on adult rudiment development see the adult rudiment ontogeny section below). The appearance of this invagination marked the *vestibule invagination stage* (Figure 11I), and its subsequent contact with the left hydrocoel defined the *vestibule contact stage* (Figure 11J). At the *vestibule contact stage*, several additional observations were made. For instance, through an immunohistochemistry assay carried out using the anti-acetylated α-tubulin antibodies, we revealed that the lumen of the hydropore, of the hydroporic canal, of the left axocoel, of the stone canal, and of the left hydrocoel (which are all components of the adult water vascular system) was packed with cilia, while the lumen of the left somatocoel, of the right axohydrocoel, and of the right somatocoel was deprived of cilia (Figures 11K–M). We further noted that the hydropore migrated from its initial left-sided position next to the esophagus (Figure 11H) towards a more medial position above the cardiac sphincter (Figure 11L). Finally, we also found that the left axocoel was significantly inflated, forming a large, coelomocyte-filled bag between the left hydrocoel and the hydropore (Figures 11K–N).

### Adult rudiment ontogeny

In echinoids, although the left axocoel, the left somatocoel, the hydropore, the hydroporic canal, and the stone canal contribute to the development of adult tissues, formation of the adult rudiment, *per se*, on the left side of the larval stomach, chiefly involves the left hydrocoel and the larval vestibule (Smith et al., 2008; Heyland and Hodin, 2014). This section thus focuses on the complex morphogenetic changes occurring at the level of the left hydrocoel and the larval vestibule during adult rudiment ontogeny, even though mention of associated structures on the left side will be made when appropriate. By contrast, the ontogeny of the right coelomic compartments will not be treated here, as these coeloms do not significantly contribute to the development of adult structures (Ziegler et al., 2009; Ezhova et al., 2014).

**FIGURE 12:**
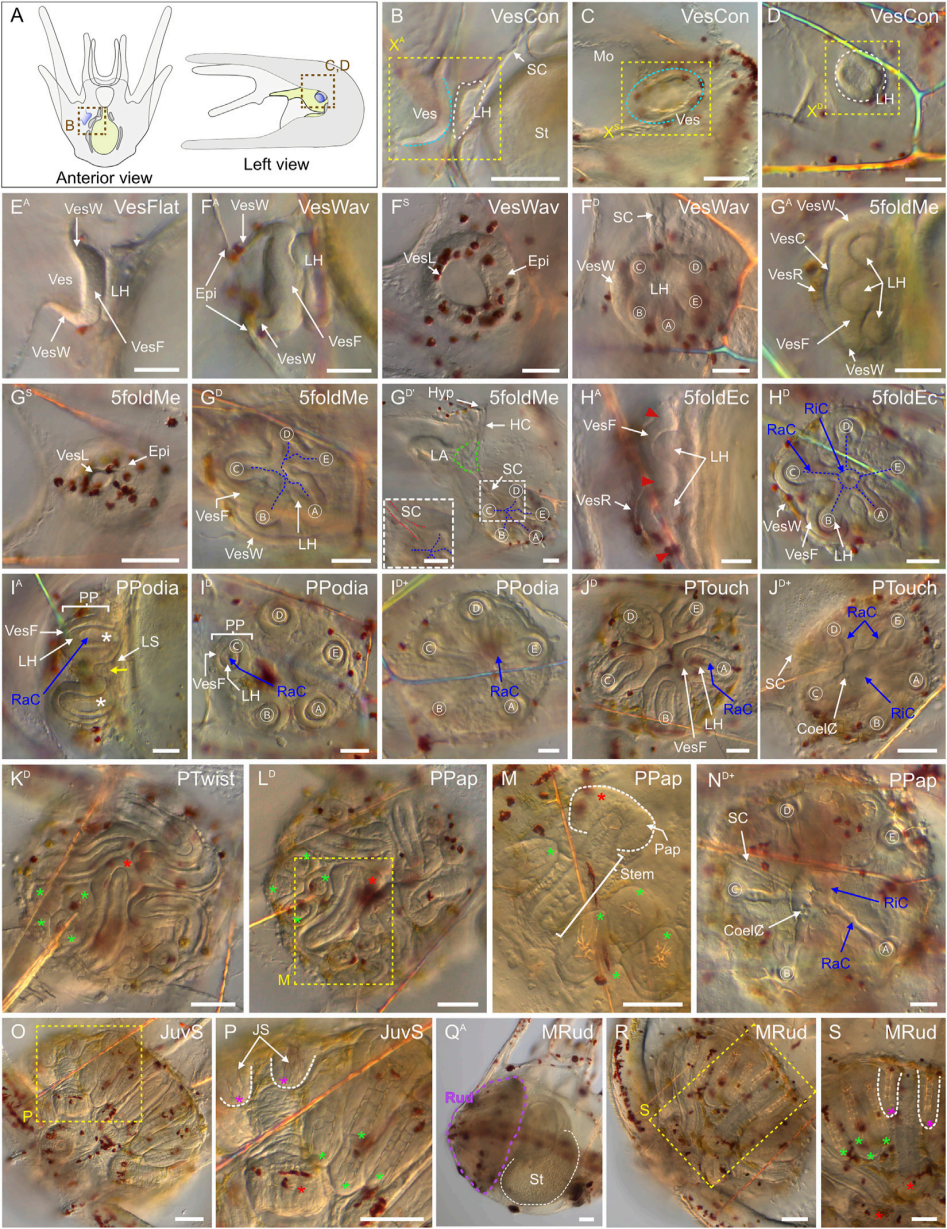
*Paracentrotus lividus* adult rudiment development under light microscopy. **(A)** Schematics of an 8-arm pluteus stage larva in anterior and left views. The vestibule (of ectodermal origin) is in blue and the larval coelomic pouch derivatives (of mesodermal origin) are in dark grey. Of note, although for simplicity we only represented an 8-arm pluteus larva, adult rudiment development actually takes place between the end of the 4-arm pluteus stage and the end of the 8-arm pluteus stage. In **(B–S)**, developmental stages are as follows: **(B–D)** vestibule contact stage (VesCon); **(E** ^**A**^ **)** vestibule flattened stage (VesFlat); **(F** ^**A**^ **–F** ^**D**^ **)** vestibule waving stage (VesWav); **(G** ^**A**^ **–G** ^**D’**^ **)** 5-fold mesoderm stage (5foldMe); **(H** ^**A**^ **–H** ^**D**^ **)** 5-fold ectoderm stage (5foldEc); **(I** ^**A**^ **–I** ^**D+**^ **)** primary podia stage (PPodia); **(J** ^**D**^ **–J** ^**D+**^ **)** primary podia touching stage (PTouch); **(K** ^**D**^ **)** primary podia twisting stage (PTwist); **(L** ^**D**^ **–N** ^**D+**^ **)** primary podia papilla stage (PPap); **(O,P)** juvenile spine stage (JuvS); **(Q** ^**A**^ **–S)** mature rudiment stage (MRud). In **(B–S)**, images were acquired using bright-field light microscopy and correspond to close-ups of the developing or developed adult rudiment. **(B–D)** Close-ups of the regions highlighted by brown boxes in **(A)**. **(E** ^**A**^ **–L** ^**D**^ **,Q** ^**A**^ **,N** ^**D+**^ **)** Close-ups following the development of the regions outlined by yellow boxes in **(B–D)** *,* with panels with superscript letters A corresponding to regions X^A^, with panels with superscript letters S corresponding to regions X^S^, and with panels with superscript letters D, D' or D+ corresponding to regions X^D^. **(M)** Close-up of the region marked by the yellow box in **(L** ^**D**^ **)**, but at a different focal plane. **(P)** Close-up of the region highlighted by the yellow box in **(O)**. **(S)** Close-up of the region outlined by the yellow box in **(R)**. In **(E** ^**A**^ **–L** ^**D**^ **,Q** ^**A**^ **,N** ^**D+**^ **)**, superscript letters refer to the position of the larva in order to observe the developing rudiment as presented, hence A **(E** ^**A**^ **,F** ^**A**^ **,G** ^**A**^ **,H** ^**A**^ **,I** ^**A**^ **,Q** ^**A**^ **)** refers to larva in anterior view (such as is **(B)**), S **(F** ^**S**^ **,G** ^**S**^ **)** refers to larva in left view, with a focal plane set at the surface of the vestibular floor (such as is **(C)**), and D **(F** ^**D**^ **,G** ^**D**^ **,G** ^**D'**^ **,H** ^**D**^ **,I** ^**D**^ **,I** ^**D+**^ **,J** ^**D**^ **,J** ^**D+**^ **, K** ^**D**^ **,L** ^**D**^ **,N** ^**D+**^ **)** refers to larva in left view, but with a focal plane positioned at a deeper level than the vestibular floor (such as is **(D)**). **(G** ^**D'**^ **)** is the same as **(G** ^**D**^ **)** but zoomed out and (**(G** ^**D'**^ **)** inset) is a close-up of the stone canal. **(I** ^**D+**^ **)** and **(J** ^**D+**^ **)** correspond to views even deeper than **(I** ^**D**^ **)** and **(J** ^**D**^ **)**, and **(N** ^**D+**^ **)** is at the same depth as **(J** ^**D+**^ **)**. In **(O,R)**, the larva is in a tilted view and in **(Q** ^**A**^ **)** a zoom of the larval apex is presented, with the larva in anterior view. In **(B,C)**, the cyan dotted line outlines the vestibule. In **(B,D)**, the white dotted line delineates the left hydrocoel. In **(F** ^**D**^ **,G** ^**D**^ **,G** ^**D'**^ **,H** ^**D**^ **,I** ^**D**^ **–J** ^**D+**^ **,N** ^**D+**^ **)**, the letters Ⓐ-Ⓔ identify the developing primary podia according to Carpenter’s nomenclature (1884). In (**(G** ^**D**^ **,G** ^**D'**^ **,G** ^**D'**^ **)** inset), blue dotted lines outline the position of the developing water vascular system. In **(G** ^**D'**^ **)**, green dotted lines highlight the left axocoel, and in (**(G** ^**D'**^ **)** inset) red dotted lines mark the stone canal. In **(H** ^**A**^ **)**, red arrowheads mark the folded vestibule epidermis. In **(H** ^**D**^ **)**, blue dotted lines mark the ring canal and radial canals of the water vascular system. In **(I** ^**A**^ **)**, the yellow arrow marks one left somatocoel bud. In **(K** ^**D**^ **,L** ^**D**^ **,M,P,S)**, green asterisks indicate the definitive spines, and red asterisks mark a primary podia papilla. In **(M)**, the white dotted line delineates a primary podia papilla. In **(P,S)**, magenta asterisks and white dotted lines mark the juvenile spines. In **(Q** ^**A**^ **)**, the magenta dotted line outlines the developed adult rudiment, and the white dotted line delineates the larval stomach. Scale bar: **(B–E** ^**A**^ **,K** ^**D**^ **–M,O–S)** 50 μm; **(F** ^**A**^ **–J** ^**D+**^ **,N** ^**D+**^ **)** 30 μm; (**(G** ^**D'**^ **)** inset) 20 µm. Coel: coelomocyte; Epi: larval epidermis; HC: hydroporic canal; Hyp: hydropore; JS: juvenile spine; LA: left axocoel; LH: left hydrocoel; LS: left somatocoel; Mo: mouth; Pap: papilla; PP: primary podium; RaC: radial canal; RiC: ring canal; Rud: adult rudiment; SC: stone canal; St: stomach: Ves: vestibule; VesC: vestibular cavity; VesF: vestibular floor; VesL: vestibular lip; VesP: vestibular pore; VesR: vestibular roof; VesW: vestibular wall.

At the *vestibule contact stage*, as mentioned above, the vestibule, composed of the thickened larval epidermis, of ectodermal origin, and the left hydrocoel, of mesodermal origin, were thus in contact. A larval anterior view revealed the C-shape of the vestibule (Figures 12A,B), while a larval left view disclosed the invagination of the vestibule (Figures 12A,C), on top of the left hydrocoel, which, at this stage, was a simple vesicle (Figures 12A,D). At the following stage, named the *vestibule flattened stage*, the epidermis of the vestibule flattened on the left hydrocoel (Figure 12E^A^). The portion of the vestibule spread over the left hydrocoel corresponded to the vestibular floor, while the remaining vestibule epidermis, still in continuity with the larval epidermis, constituted the vestibular walls (Figure 12E^A^). Thereafter, the larval epidermis started to close above the vestibular cavity, and the apical surface of the vestibular floor, in contact with the left hydrocoel, started to show folds (Figure 12F^A^). This marked the *vestibule waving stage*. Closure of the larval epidermis induced the emergence of a hole on the larval epidermis, delineated by the vestibular lip (Figure 12F^S^). Furthermore, at the level of the left hydrocoel, a critical morphogenetic reorganization took place at this stage. The left hydrocoel began folding at the *vestibule waving stage*, thereby taking a flower-like shape composed of five lobes and connected, at its center, to the left axocoel by the stone canal (Figure 12F^D^). This reorganization marked the first stage in the acquisition of the adult pentaradial symmetry and highlighted the central role of the left hydrocoel (and hence of the mesodermal coelomic tissue) in the switch of animal body plan symmetry in *P. lividus*. The five lobes prefigured already the five ambulacra of the adult, and their respective position relative to that of the stone canal indicated that their identity was already defined, with the stone canal facing ambulacrum A and being located between ambulacra C and D (Supplementary Figure S1). Of note, we here used a regular clockwise Carpenter’s nomenclature to identify the five lobes, taking into consideration that we observed the rudiment from the left side of the larva, which corresponds to the future oral side of the adult.

The next developmental stage, called the *5-fold mesoderm stage*, was characterized by the protrusion of the five lobes of the left hydrocoel into the vestibular floor (Figure 12G^A^), which otherwise did not show any sign of folding or of pentaradial symmetry on its basal side (Figure 12G^A^). This stage was observed during (Figure 12G^S^) or after (Figure 12G^A^) closure of the vestibular lip. Closure of the vestibular lip was often completed by this stage, although, in some larvae, a small bridge between the larval ectoderm and the vestibular roof remained until later stages. Nevertheless, by the *5-fold mesoderm stage*, the vestibule was always composed of a roof, a floor, and walls, all of which were surrounding a cavity (Figure 12G^A^). In addition, by this stage, the left hydrocoel had always undergone additional morphological changes. For instance, a lumen expanded within each of the five lobes (Figure 12G^D^). These five lumens represented the anlage of the five radial canals of the adult water vascular system. Moreover, at the center of the left hydrocoel, the anlage of the future ring canal, which will later connect the five radial canals, was discernable (Figure 12G^D^). However, at the *5-fold-mesoderm stage*, the ring canal was still C-shaped (Figure 12G^D^) and thus not yet closed. Furthermore, at the *5-fold-mesoderm stage*, each of the lumen of the five lobes of the left hydrocoel was a direct prolongation of the lumen of the stone canal, which was still connected to the left axocoel, and therefore to the external environment through the hydroporic canal and the hydropore (Figure 12G^D'^). At this stage, the adult rudiment was thus already susceptive of experiencing a seawater flow.

Subsequently, the *5-fold ectoderm stage* was characterized by the complete folding of the vestibular floor onto the underlying left hydrocoel (Figure 12H^A^). At this stage, both the apical and basal surfaces of the vestibular floor were folded (Figure 12H^A^), and the vestibular floor thus exhibited, such as the left hydrocoel, a pentaradial symmetry (Figure 12H^D^). At this stage, within the hydrocoel, the extremities of the C-shaped ring canal also fused into a proper ring, and, within the lobes, the radial canals became more conspicuous (Figure 12H^D^). Thereafter, the five lobes and their associated inner radial canals extended even further, pushing the vestibular floor into the vestibular cavity (Figure 12I^A^). As the lobes elongated, they became recognizable as the primary podia, another component of the water vascular system (MacBride, 1918). Accordingly, this stage was named the *primary podia stage*. The primary podia were composed of two tissue layers: on the outside, the vestibular floor, of ectodermal origin, and on the inside, the left hydrocoel, of mesodermal origin and delineating the radial canals (Figures 12I^A^–I^D+^). By the *primary podia stage*, the left somatocoel also extended anteriorly along the stomach and underneath the left hydrocoel and adopted a pentaradial symmetry. At this stage, the left somatocoel was discernable as five buds alternating with the five primary podia (Figure 12I^A^).

As developmental proceeded, the primary podia continued to extend into the vestibular cavity. As they became longer, they progressively bent inward into the cavity, ultimately touching each other at their tips (Figure 12J^D^). This marked the *primary podia touching stage*. Optical sections through the podia and the rudiment at this stage revealed the radial canals at the center of the podia (Figures 12J^D^,J^D+^) as well as the ring canal, which was now fully formed and functional and started to contain coelomocytes (Figure 12J^D+^). Subsequently, the continuous growth of the podia forced them to bend even further and to twist within the vestibular cavity (Figure 12K^D^). This marked the *primary podia twisting stage*. At this stage, the epidermis at the tip of the primary podia started to thicken and to adopt an arrow-like shape (Figure 12K^D^). An additional characteristic of this stage was the emergence of five groups of four spine sacs, in alternation with the five primary podia (Figure 12K^D^), hence resulting in a total of twenty spine sacs within the rudiment.

At the following developmental stage, called the *primary podia papilla stage*, the most easily recognizable morphological change was the appearance, at the tip of the primary podia, of a fully formed papilla (Figures 12L^D^,M). At this stage, the primary podia were thus composed of a stem and a papilla (Figure 12M), the latter being a flat, discoid structure used in the adult for adhesive attachment as well as for mechano-, chemo-, and photo-reception (Ullrich-Lüter et al., 2011). By this stage, each spine sac also elongated, giving rise to the anlage of the so-called definitive (or adult) spines (Figure 12M), as they will be maintained through metamorphosis and in adult life (Hyman, 1955; Emlet, 2010). In addition, at the level of the water vascular system, a growing number of coelomocytes became visible at the *primary podia papilla stage*, both in the ring canal and in the radial canals (Figure 12N^D+^). A water flow was now clearly visible, with external water being pumped into the water vascular system by the left axocoel (Supplementary Video S2). Coelomocytes were thus carried back and forth by the water flow (Supplementary Video S3), and the podia were mobile within the vestibular cavity as they were injected with water (Supplementary Video S4).

The next stage was characterized by the emergence of a new set of spines called the juvenile spines (Figures 12O,P), which correspond to spines that will be maintained through metamorphosis, but will not persist in adult life (Emlet, 2010). The appearance of these new spines marked the *juvenile spine stage*. The juvenile spines formed as a pair at the base of each of the five primary podia, with one juvenile spine on each side of the primary podium stems (Figures 12O,P). This resulted in a total of ten juvenile spines within the rudiment. At the *juvenile spine stage*, the podia also continued to expand, leading to the shrinking of their stem, which developed an accordion-like aspect (Figure 12P). The definitive spines also extended (Figure 12P), and the rudiment significantly increased its overall size (Figure 12O). The last stage of rudiment development corresponded to the *mature rudiment stage*. At this stage, the rudiment had a diameter of about 350 µm. It was bigger in size than the larval stomach and occupied almost the complete left side of the larva (Figure 12Q^A^). At this stage, the juvenile spines elongated and reached about half the size of the definitive spines (Figures 12R,S). Of note, although the previous steps of rudiment development were largely heterochronous with respect to the development of the larva, the *mature rudiment stage* was exclusively observed once the larvae were fully developed, i.e., by the end of the *8-arm pluteus stage*.

### Skeletogenesis and myogenesis in the adult rudiment

**FIGURE 13:**
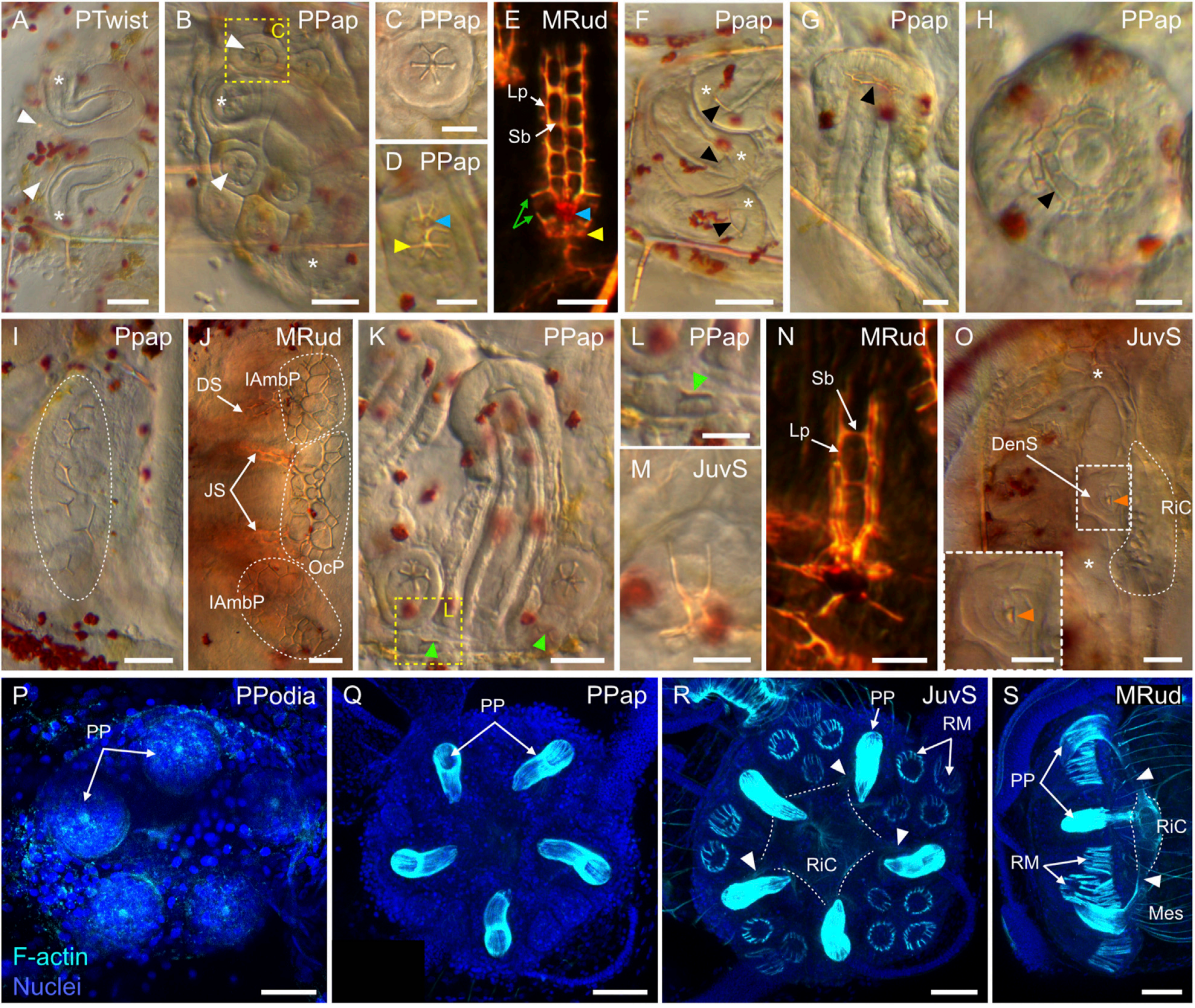
Skeletogenesis and myogenesis in the *Paracentrotus lividus* adult rudiment. Developmental stages are as follows: **(A)** primary podia twisting stage (PTwist); **(B–D,F–I,K,L,Q)** primary podia papilla stage (PPap); **(E,J,N,S)** mature rudiment stage (MRud); **(M,O,R)** juvenile spine stage (JuvS); **(P)** primary podia stage (PPodia). In **(A–D,F–M,O)**, images were acquired using bright-field light microscopy. In **(E,N)**, images were obtained using polarized light. In **(P–S)**, images are maximum intensity projections of confocal z-stacks of larvae co-labeled for F-actin (muscles; cyan) and DNA (nuclei; blue), and they correspond to projections of the entire rudiment. **(A–E)** Close-ups of the skeletal element associated with the definitive spines in top, oral view in **(A–D)** and in lateral view in **(E)**. **(C)** Close-up of the region outlined by the yellow box in **(B)**. **(F–H)** Close-ups of primary podia, in lateral view in **(F,G)** and in papilla view in **(H)**. **(I,J)** Close-up of the anlage of the future adult skeletal shell at the surface of the developing adult rudiment, which is in side view. **(K–N)** Close-up of the skeletal element associated with the juvenile spines in top, oral view in **(K–M)** and in lateral view in **(N)**. **(L)** Close-up of the region highlighted by the yellow box in **(K)**. **(O)** Close-up of the ring canal and of a dental sac in an adult rudiment in side view. (**(O)** inset) Close-up of the dental sac. **(P–S)** Close-ups of the developing adult rudiment in oral, deep view in **(P–R)** and in side view in **(S)**. In **(A,B)**, white arrowheads highlight the developing skeletal spicules of definitive spines. In **(A,B,F,O)**, white asterisks mark the primary podia. In **(D,E)**, the yellow arrowhead indicates the hexaradiate spicule of a definitive spine, and the blue arrowhead marks the hexagonal ring. In **(E)**, green arrows highlight additional skeletal branches emerging from either the hexaradiate spicule or the hexagonal ring. In **(F–H)**, black arrowheads highlight developing primary podia skeletal disks. In **(I,J)**, white dotted lines delineate the anlage of the future adult skeletal shell in **(I)**, and the developing ocular and interambulacral plates in **(J)**. In **(K,L)**, green arrowheads mark the developing triradiate spicules of juvenile spines. In (**(O)**, **(O)** inset), orange arrowheads highlight a primordium of a developing tooth located within a dental sac. In **(O,R,S)**, white dotted lines outline the ring canal. In **(R,S)**, white arrowheads mark the muscles associated with the radial canals. Scale bar: **(A,B,F)** 50 μm; **(C,D,L,M)** 12.5 µm; **(E,K,N,P–S)** 25 μm; **(G,H)** 20 μm; **(I,J,O)** 15 μm; (**(O)** inset) 7.5 µm. DenS: dental sac; DS: definitive spine; IAmbP: interambulacral plate; JS: juvenile spine; Lp: longitudinal processes; Mes: mesentery; OcP: ocular plate; PP: primary podium; RiC: ring canal; RM: ring muscle; Sb: stereomic bridge.

During rudiment development, several rudiment-specific skeletal structures and muscles were also formed. Skeletogenesis in the rudiment usually started at the *primary podia twisting stage* with the emergence of spicules associated with the definitive spines (Figure 13A). As in the larva, most skeletal elements formed in the rudiment from an initial crystal that subsequently developed smooth rods along each of its a-axes. This was the case, for instance, for the definitive spines (Figure 13A), but not every rudiment skeletal piece emerged from a new, independent crystal. Some branched out indeed directly from neighboring larval rods. In the case of the definitive spines, the spicules that emerged from the crystals were moreover peculiar, in that they displayed a unique hexaradiate structure (i.e., a structure with six apices instead of three, as seen for all other larval or rudiment spicules) (Figures 13B,C). Development of the hexaradiate spicules was characterized by the emergence, from the initial crystal, of six smooth skeletal rods that grew laterally, in the same plane, for a short distance (Figure 13C). A new branch then extended perpendicular to the first ones from the center of the hexaradiate spicule (Figure 13D). From the top of this perpendicular branch, three processes grew outward, generating a three-rayed star, from which an additional hexagon formed, parallel to the initial hexaradiate spicule, but smaller in diameter (Figures 13D,E). From this second hexagon, six branches extended longitudinally inside the spine sacs and towards the vestibular cavity (Figure 13E). These branches constituted the endoskeleton of the elongating definitive spines. They were periodically joined by regularly spaced stereomic bridges, thereby creating a long, fenestrated skeletal element (Figure 13E).

The next skeletal elements to develop were those associated with the primary podia, referred to as the primary podia skeletal disks (Figures 13F,G) (Formery et al., 2021). The primary podia skeletal disks first emerged between the *primary podia twisting* and the *primary podia papilla stages*. They initially appeared as a single, triradiate spicule located at the base of the papilla. Then, two of the three apices of the spicule grew laterally, creating an arch (Figure 13F). The two growing apices eventually fused and gave rise to a disk (Figure 13G), before, due to subsequent branching events, the architecture of the disk became even more sophisticated (Figure 13H). Between the *primary podia twisting* and the *primary podia papilla stages*, additional triradiate spicules further started to form at the periphery of the rudiment, which constituted the first components of the future adult test (Figure 13I). These spicules subsequently underwent extensive branching and resulted, at the *mature rudiment stage*, in the establishment of very distinctive endoskeletal plates (Figure 13J). The names of these distinct endoskeletal plates depends on their position within the rudiment (Gordon, 1926). For instance, the plates forming at the base of the primary podia stem and the juvenile spines are referred to as the ocular plates, while these developing at the level of the definitive spines are the interambulacral plates.

At the *primary podia papilla stage*, the skeletal elements holding the juvenile spines also started to appear. Initially, the spicules at the origin of these elements arose as triradiate crystals (Figures 13K,L), even though most of them subsequently developed into tetraradiate spines (Figure 13N). The transition of an initial triradiate crystal to an elongated tetraradiate spine was a multi-step process. First, each of the three apices of the initial triradiate crystal grew laterally for a short distance. Then, halfway along each of the three smooth rods, a new branch emerged, which grew perpendicular to the initial plane of the triradiate spicule, i.e., towards the vestibular cavity (Figure 13M). Of these perpendicular branches, two usually continued to extend upwards, while the third one divided into two distinct branches before resuming its upwards growth (Figure 13M). As a result, most juvenile spines were composed of four longitudinal processes, which, like in definitive spines, were connected by regularly spaced stereomic bridges (Figure 13N). The last skeletogenic event that we observed in the rudiment was the development of skeletal structures associated with the future masticatory apparatus. This event took place at the *juvenile spine stage*, in the five buds formed by the left somatocoel and located between the primary podia (Figure 13O). These buds correspond to the dental sacs (MacBride, 1903) and, at the center of each one of them, we identified a calcite crystal corresponding to the primordium of the future adult teeth (Figure 13O).

In addition to the skeletal structures, we also characterized the muscles that developed within the adult rudiment, carrying out an immunohistochemistry assay for F-actin. Myogenesis started in the rudiment at the *primary podia stage* with an accumulation of F-actin at the center of each emerging primary podium, revealing the presence of growing muscle fibers (Figure 13P). By the *primary podia papilla stage*, we were able to confirm the presence of these muscle fibers within each of the five primary podia as long muscle strands outlining the water vascular system (Figure 13Q). At the *juvenile spine* and *mature rudiment stages*, additional muscle fibers were found in other compartments of the water vascular system as well as at the level of the definitive spines (Figures 13R,S). At the center of the rudiment, our F-actin staining highlighted muscle fibers associated with the ring canal as well as with the radial canals, which extended from the ring canal to the stem of the primary podia (Figures 13R,S). In addition, at the base of each definitive spine, several muscle fibers were detected that were arranged in a circular manner (Figures 13R,S) and constituted the so-called ring muscles (Formery et al., 2021).

### Development of complementary adult structures: The pedicellariae and the genital plates

**FIGURE 14:**
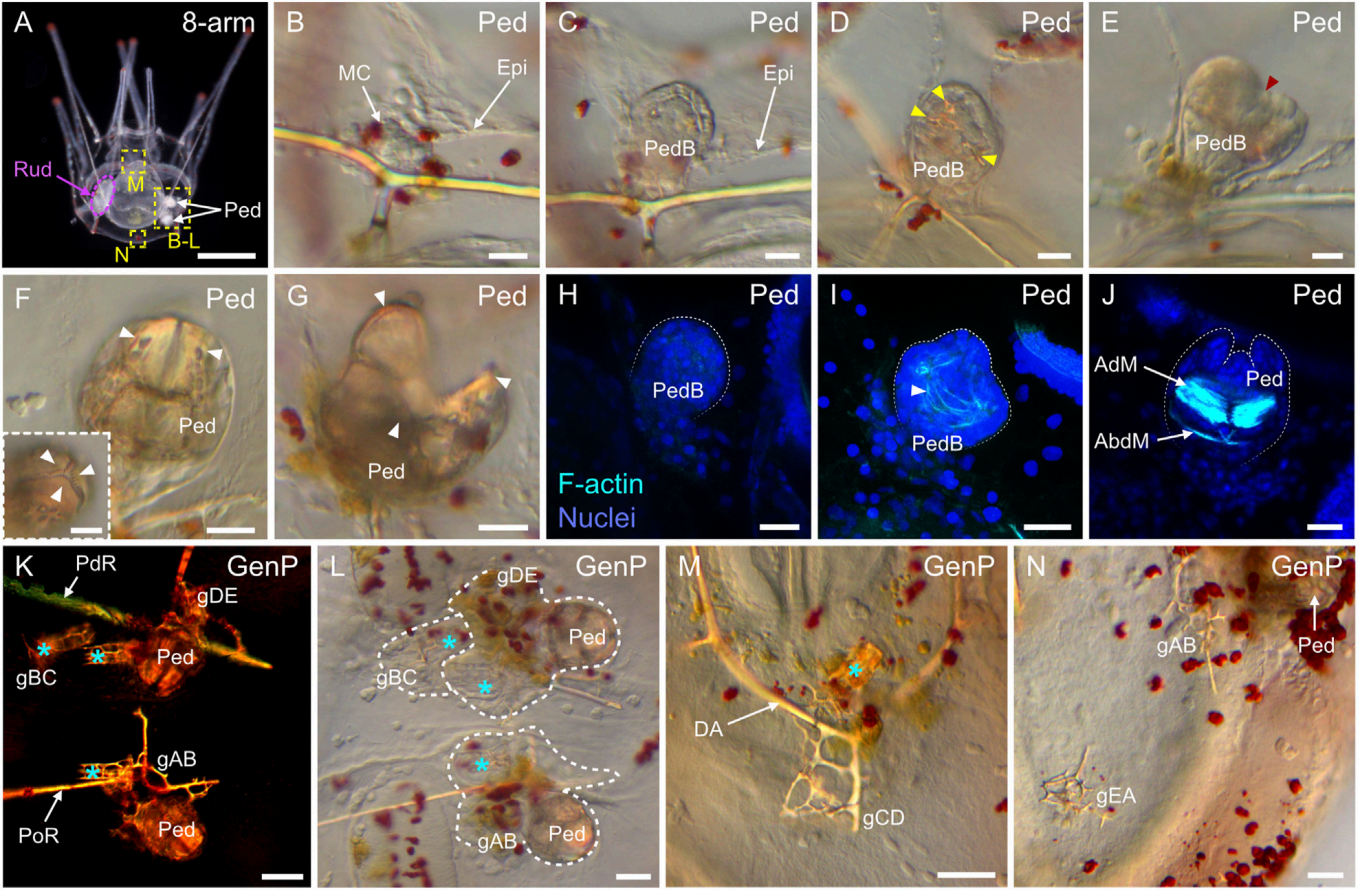
Development of the pedicellariae and the genital plates in *Paracentrotus lividus* larvae. In **(A–N)**, images are from larvae at the 8-arm pluteus stage (8-arm). In **(A)**, the image was acquired using dark-field light microscopy. In **(B–G,L–N)**, images were obtained using bright-field light microscopy. In **(H–J)**, images are maximum intensity projections of confocal z-stacks of larvae co-labeled for F-actin (muscles; cyan) and DNA (nuclei; blue), and they correspond to projections of the entire pedicellaria. In **(K)**, the image was taken using polarized light to highlight the skeletal elements. In **(A)**, the larva is in anterior view, with the ventral side up and the left side to the left. **(B–N)** Close-ups of the regions outlined by yellow boxes in **(A)**. **(B–J)** Close-ups of pedicellariae (Ped). **(K–N)** Close-ups of genital plates (GenP). In **(A)**, the purple dotted line delineates the adult rudiment on the left side of the larva. In **(D)**, yellow arrowheads indicate the skeletal elements developing inside the pedicellaria bud. In **(E)**, the red arrowhead shows the individualization of the three lobes within the bud. In (**(F)**, **(F)** inset, **(G)**), white arrowheads mark the three jaws of the pedicellaria, which can be either closed like in **(F)** and (**(F)** inset) or open like in **(G)**. In **(H–J)**, white dotted lines outline the pedicellaria bud in **(H–I)** or the pedicellaria jaws in **(J)**. In **(I)**, the white arrowhead points to the first muscle fibers appearing within a pedicellaria bud. In **(K–M)**, cyan asterisks indicate the position of juvenile spines associated with the genital plates. In **(L)**, white dotted lines delineate the genital plates and their associated juvenile spines developing in the vicinity of pedicellariae. Scale bar: **(A)** 200 μm; (**(B–G)**, **(F)** inset) 15 μm; **(H–J,K–M)** 30 μm; **(N)** 50 µm. AdM: adductor muscle; AbdM: abductor muscle; DA: dorsal arch; Epi: larval epidermis; gAB: genital plate AB; gBC: genital plate BC; gCD: genital plate CD; gDE: genital plate DE; gEA: genital plate EA; GenP: genital plate; MC: mesenchyme cell; PdR: posterodorsal rod; Ped: pedicellaria; PedB: pedicellaria bud; PoR: postoral rod; Rud: adult rudiment.

Apart from the rudiment, developing on the left side of the larval digestive tract, some additional adult structures further developed in the larva that persisted after metamorphosis. These structures included the pedicellariae and the genital plates as well as additional juvenile spines associated with the genital plates. The pedicellariae and three of the five genital plates developed on the right side of the larva (and thus on the opposite side of the rudiment), while the two other genital plates developed, respectively, at the level of the dorsal arch and near the apex (Figure 14A). All these structures usually developed once the larva had reached the *8-arm pluteus stage* and after coelomogenesis started, but their ontogeny was not linked to that of the larva, the coeloms, or the rudiment. As such, their development was thus not included in the general staging scheme or in the coeloms and rudiment development subsection in Figure 3.

The pedicellariae are a special type of appendages found in asteroids and echinoids, which are used to protect the animal (Hyman, 1955; Peters and Campbell, 1987). In *P. lividus*, we observed the development of one to four pedicellariae per larva, although, in some rare cases, no pedicellariae were formed. The development of the pedicellariae started by a condensation of mesenchymal cells below the larval epidermis, at the level of the branching points between the right posterodorsal and the right dorsoventral connecting rods and/or between the right postoral and the right dorsoventral connecting rods (Figure 14B). Soon thereafter, the condensed cells formed a well-differentiated bud protruding outside the larval epidermis (Figure 14C), with skeletal elements starting to develop inside the bud shortly after it was formed (Figure 14D). Subsequently, the bud segregated into three lobes (Figure 14E), which progressively differentiated into three calcified jaws, with an indented extremity and an inner skeleton characterized by a complex fenestrated architecture (Figures 14F,G). The jaws rapidly showed motility, being capable of opening and closing (Figures 14F,G; Supplementary Video S5). They were thus already functional in the larva. A labeling for F-actin moreover revealed that the pedicellariae had their own muscular system (Figures 14H–J). The first muscle fibers appeared in the buds of the pedicellariae when the three lobes started to individualize (Figures 14H,I). Then, once the lobes were fully developed, the pedicellariae were characterized by two sets of three muscles, connecting the jaws and allowing them to open and close (Figure 14J). These muscles are called the large adductor muscles, located near the indented extremities, and the thin abductor muscles, located closer to the stem (Peters and Campbell, 1987).

The genital plates are specific endoskeletal plates that, in the adult, form the most aboral part of the test and bear the gonopores, hence their name (see Figure 1E) (Hyman, 1955). According to Carpenter’s nomenclature, the genital plates are identified as AB, BC, CD, DE, and EA due to their interambulacral position in the adult (Supplementary Figure S1) (Carpenter, 1884). In the larva, the genital plates AB, CD, and DE were the first to develop, and they all formed from the branching of a larval rod (Figures 14K–M). The genital plates AB and DE developed from an outgrowth, respectively, of the right postoral and the right posterodorsal rods. They were hence often found at the base of a pedicellaria (Figures 14K,L). The genital plate CD developed at the branching point of the dorsal arch (Figure 14M). It was thus located next to the hydropore, where it will differentiate into the madreporite of the adult. The next genital plate to develop was the genital plate BC, which, unlike its counterparts, did not form from a larval skeletal rod but from a *de novo* triradiate spicule positioned in the right lateral field near the genital plate DE (Figures 14K,L). Finally, the last genital plate to form was the genital plate EA (Figure 14N), which emerged near the larval apex at the *mature rudiment stage*. Since it formed significantly later than the other genital plates, the genital plate EA was always the smallest. By the time of metamorphosis, all genital plates, including the genital plate EA, were further supporting one or two juvenile spines (Figures 14K–M). Therefore, by the time of metamorphosis, a total of fifteen to nineteen juvenile spines were discernable on the larva: ten associated with the rudiment and five to nine associated with the genital plates.

### *Competent pluteus stage* and metamorphosis

**FIGURE 15:**
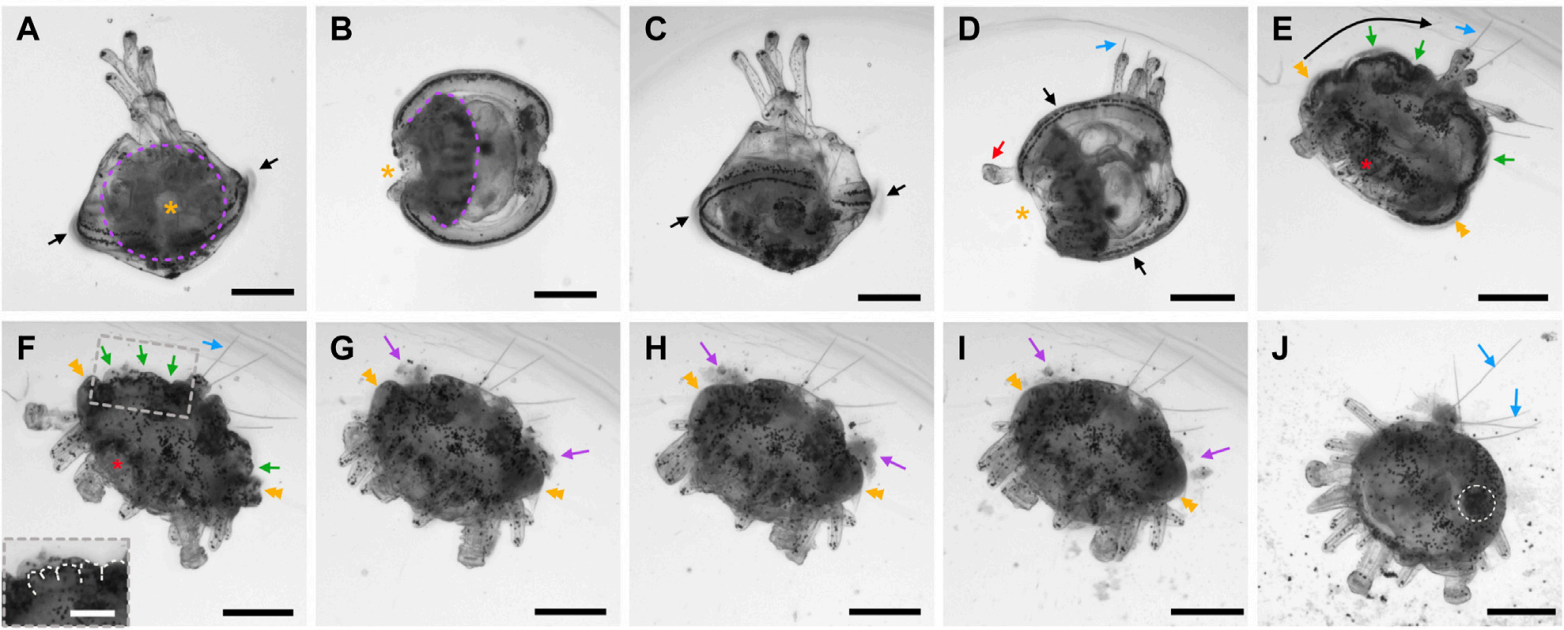
The competent pluteus stage and metamorphosis in *Paracentrotus lividus*. In **(A–J)**, images were extracted from a movie recorded using bright-field light microscopy. In **(A)**, the larva is in left view, with the ventral side up. In **(B)**, the larva is in dorsal view, with the rudiment on the left. In **(C)**, the larva is in right view, with the ventral side up. In **(D)**, the larva is in dorsal, tilted view, with the rudiment on the left. In **(E–J)**, the developing juvenile is in side view, with the oral surface towards the bottom left corner. (**(F)** inset) Close-up of the folds at the level of the larval body epidermis. In **(A,B)**, purple dotted lines outline the adult rudiment. In **(A,B,D)**, the orange asterisk highlights the vestibular pore. In **(A,C,D)**, black arrows point to the larval epaulettes. In **(D)**, the red arrow marks a protruding primary podium. In **(D–F,J)**, blue arrows highlight the larval arms and rods. In **(E)**, the long black arrow illustrates the movement of the larval body epidermis from the vestibular pore towards the larval arms and pedicellariae. In **(E,F)**, green arrows highlight folds of the larval body epidermis. In **(E–I)**, orange double arrowheads mark the bulge forming around the juvenile appendages by contraction and eversion of the vestibular lips and walls. In (**(F)** inset), white dotted lines outline folds of the larval body epidermis. In **(G–I)**, purple arrows point to delaminating cells. In **(J)**, the white dotted line encircles a pedicellaria. Scale bar: **(A–H)** 200 μm; (**(F)** inset) 100 µm.

Once the larvae were at the *8-arm pluteus stage*, the pedicellariae and the genital plates were formed, and the rudiment was at the *mature rudiment stage*, the larvae were at the so-called *competent pluteus stage* (Figures 15A,B). In echinoids, competency means that the larva is morphologically ready to undergo metamorphosis, although it relies on the detection of appropriate environmental cues to trigger this process (Strathmann, 1978; Burke, 1980). Under our culture conditions, the *competent pluteus stage* was reached at around 30 dpf, as assessed both morphologically and by the observation of spontaneous metamorphosis in our culture vessels. However, all larvae of a given culture vessel never underwent metamorphosis simultaneously. This was primarily due to the heterochrony of rudiment, pedicellaria, and genital plate development, but potentially also because some larvae detected the required environmental cues earlier than others. Thus, the duration of the *competent pluteus stage* was variable within a given culture, with the initiation of metamorphosis by some larvae happening up to a couple of weeks after the first larvae of that culture underwent metamorphosis.

Larvae at the *competent pluteus stage* were characterized by subtle morphological features. First, the rudiment was at the *mature rudiment stage* and occupied all of the larval body on the left side of the stomach (Figures 15A,B). Second, a hole was present in the left larval epidermis, in front of the rudiment (Figure 15B). This hole, identified as the vestibular pore (Smith et al., 2008), arose either from the bridge between the larval epidermis and the vestibular roof or resulted from a *de novo* fusion of these two tissues (data not shown). In addition, at the *competent pluteus stage*, the larvae displayed some behavioral changes. First, ciliary beating of the epaulettes (Figure 15C) was reduced periodically, and the larvae thus sank to the bottom of the culture vessel (data not shown). Second, while at the bottom of the culture vessel, one or more primary podia extended through the vestibular pore and probed the substrate, presumably in search of an adequate environment for settlement (Figure 15D). This behavior was observed during the *competent pluteus stage* but also upon initiation of the process of metamorphosis, which was always marked by the shrinking of the ectoderm at the level of the larval arms (Figure 15D).

The process of metamorphosis, leading to the transformation of the larvae into benthic juveniles, took between 1 and 2 h under our culture conditions (Figures 15D–J; Supplementary Video S6). Its onset was always characterized by a specific sequence of fast and irreversible morphological events. First, the larva stopped swimming and the epidermis of the larval arms shrank, leaving behind the skeletal rods (Figure 15D; Supplementary Video S6). Then, the vestibular pore opened up, thereby creating a bulge on the edge of the vestibular pore (constituted of the vestibular lip and part of the vestibular walls) and pushing upwards the epidermis of the larval body towards the ventral and right sides of the larva (Figure 15E; Supplementary Video S6). Concomitantly, the podia and spines of the rudiment, inside the vestibular cavity, started to move very actively and to spread laterally, leading to an additional enlargement of the vestibular pore, to the protrusion of the podia and spines outside of the vestibular cavity, and to the continued condensation of the larval body epidermis on the ventral and right sides of the larva (Figure 15F; Supplementary Video S6). The accumulation of the larval epidermal tissue resulted in the formation of noticeable folds on the ventral and right sides of the larva (Figures 15F,F inset). In addition, at the level of the vestibular pore, the bulge eventually everted, spreading away from the podia and the spines, towards the ventral and right sides of the larva (Figure 15F). Thereafter, a large number of cells started to delaminate from the ventral and right regions of the larval epidermis (Figures 15G–I; Supplementary Video S6). While the cells delaminated, the folds of the larval epidermis gradually disappeared, the bulge resorbed, and the epidermis stretched out (Figures 15G–I). Once cell delamination stopped, the pentaradial juvenile appeared and started to crawl on the substrate (Figure 15J). It was covered by an epidermis punctuated with red-pigmented cells and still featured, on the surface facing away from the substrate, remnants of the larval arm rods (Figure 15J). It is worth noting that metamorphosis led to a radical modification of the animal body axes: the left side of the larva became the oral side of the juvenile (facing toward the substrate) and the right side of larva became the aboral side of the juvenile (facing away from the substrate) (Supplementary Figure S1).

### Juvenile stages

**FIGURE 16:**
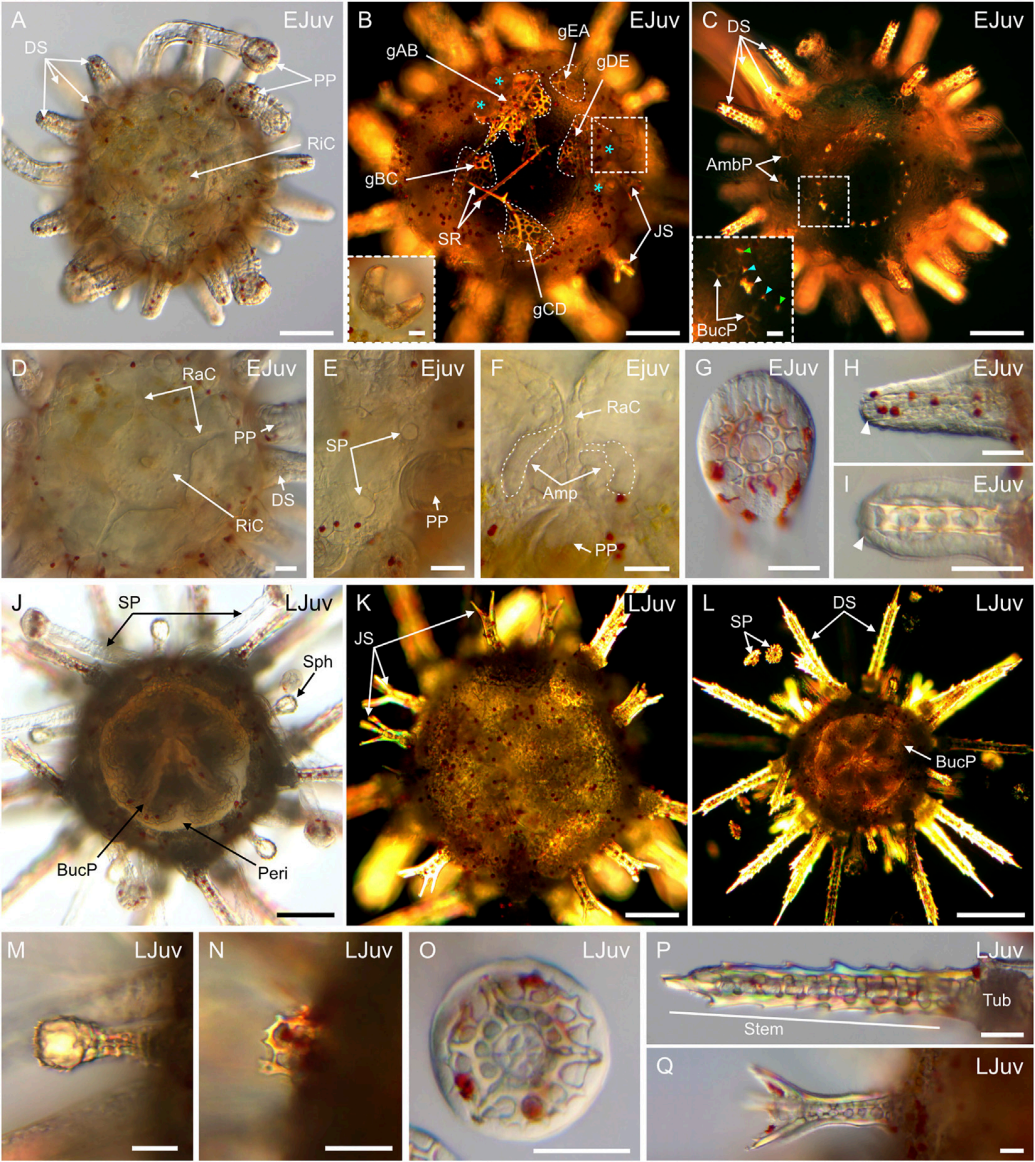
Anatomy of early and late juveniles of *Paracentrotus lividus*. Developmental stages are as follows: **(A–I)** early juvenile stage, 1 day post-metamorphosis (EJuv); **(J–Q)** late juvenile stage, 8 days post-metamorphosis (LJuv). In **(A,D–J,M–Q)**, images were acquired using bright-field light microscopy. In **(B,C,K,L)**, images were obtained using polarized light microscopy. In **(A–D,J–L)**, images correspond to specimens in oral view in **(A,C,D,J,L)** and in aboral view in **(B,K)**. (**(B)** inset) Close-up of an opened pedicellaria. (**(C)** inset) Close-up of the skeletal elements constituting the primordia of the adult masticatory apparatus. **(D)** Close-up of the ring and radial canals of the water vascular system. **(E)** Close-up of a pair of growing secondary podia located underneath a primary podium. **(F)** Close-up of the ampullae formed at the proximal tip of a radial canal. **(G)** Close-up of the skeletal disk within the papilla of a primary podium. **(H)** Close-up of a developing definitive spine. **(I)** Close-up of a developing juvenile spine. **(M)** Close-up of a sphaeridium. **(N)** Close-up of a degenerated primary podium. **(O)** Close-up of the skeletal disk within the papilla of a secondary podium. **(P)** Close-up of a fully formed definitive spine. **(Q)** Close-up of a fully formed juvenile spine. In **(B)**, cyan asterisks mark the pedicellariae present on the aboral surface of the juvenile, and white dotted lines delineate the genital plates. In (**(C)** inset), the white arrowhead indicates a tooth primordium, cyan arrowheads highlight primordia of the hemipyramids, and green arrowheads mark primordia of the epiphyses. In **(F)**, white dotted lines outline two ampullae formed at the proximal tip of a radial canal. In **(H,I)**, white arrowheads indicate the apex of a growing definitive spine in **(H)** and of a growing juvenile spine in **(I)**. Scale bar: **(A–C,J–L)** 100 μm; (**(B)** inset, **(C)** inset, **(D–I,M–Q)**) 30 µm. Amp: ampulla; AmbP: ambulacral plate; BucP: buccal plate; DS: definitive spine; gAB: genital plate AB; gBC: genital plate BC; gCD: genital plate CD; gDE: genital plate DE; gEA: genital plate EA; JS: juvenile spine; Peri: peristome; PP: primary podium; RaC: radial canal; RiC: ring canal; SP: secondary podium; Sph: sphaeridium; SR: skeletal rod; Tub: tubercule.

Following metamorphosis, the animal entered the adult period, i.e., living in a different environment (from pelagic to benthic), with a different lifestyle (from swimming and particle feeding to crawling and grazing), and exhibiting a completely different body organization (from bilateral to pentaradial symmetry). Because of the heterochrony observed during the *competent pluteus stage*, with respect to the timing of metamorphosis, a certain level of anatomical heterogeneity was observed among post-metamorphic juveniles. This was particularly obvious when comparing, for instance, the size of the skeletal plates or the length of the spines between different juveniles, as these structures continued to develop in the rudiment and the larva upon acquisition of competency and before metamorphosis. Despite these differences, the overall anatomy of the juveniles was very similar, and 1 day post-metamorphosis (dpm) all juveniles resembled miniature versions of *P. lividus* adults with spherical bodies bearing different appendages, including podia, spines, and pedicellariae (Figure 16A).

A major difference to the adult was that the post-metamorphic juvenile, at 1 dpm, lacked a fully formed mouth and anus and thus a functional digestive system. The juvenile hence exclusively relied on internal sources of energy, and this endotrophic state was the main characteristic of the *early juvenile stage*. At 1 dpm, the juvenile was further characterized by the lack of a complete skeletal test. The test was only composed of the endoskeletal plates inherited from the rudiment and the larva, which were not yet large enough to fuse and cover the entire animal (Figures 16B,C). For instance, on the aboral side, the five genital plates were present (Figure 16B), but they were still independent from one another as well as from the ocular and the interambulacral plates. Nonetheless, 1 dpm, the genital plates (AB, BC, CD, DE, and EA) were already exhibiting a characteristic circular arrangement corresponding to Carpenter’s nomenclature (Figure 16B). In addition, the genital plates AB, CD, and DE, which developed from the larval rods, were still generally marked by the presence of remnants of their respective rods (Figure 16B). On the oral side, the buccal plates and the newly developing ambulacral plates were also still, 1 dpm, simple triradiate spicules with only a few branches (Figure 16C). In the center of the oral side, 25 small ossicles were further distinguishable, which were arranged in a circular manner (Figure 16C). These small ossicles corresponded to the primordia of the skeletal pieces of the future masticatory apparatus of the adult, i.e., Aristotle’s lantern. The five most conspicuous of these ossicles corresponded to the tooth primordia (Figure 16C inset). These were flanked on both sides by a pair of developing hemipyramids and a pair of developing epiphyses (Figure 16C inset), two elements that will later form the skeletal structure supporting the teeth (Vellutini and Migotto, 2010; Ziegler et al., 2012).

Concerning the appendages, the 1 dpm juvenile inherited from the larva: the pedicellariae, the primary podia and their related water vascular system, and the definitive and juvenile spines. On the aboral surface of the juvenile, the pedicellariae remained attached to the genital plates and were functional (Figure 16B). The water vascular system was still composed of a functional ring canal and five radial canals, enabling the extension and retraction of the five primary podia (Figures 16A,D). At 1 dpm, the juveniles were also characterized by a set of ten growing secondary podia, located, in pairs, underneath the primary podia (Figure 16E), and a set of ten ampullae, emerging, in pairs, on each side of the proximal extremity of the radial canals (Figure 16F). At this stage, the primary podia further exhibited a complete skeletal disk formed, as indicated above, from a single spicule (Figure 16G). The definitive and juvenile spines, inherited from both the rudiment and the larva, were also still present in 1 dpm juveniles, although they usually featured rounded tips (Figures 16A–C,H,I), indicating that they were not yet fully developed.

Following metamorphosis, the development of the animal did not stop. The juveniles entered a new phase of gradual reorganization of both their inner and outer anatomy. This reorganization included, for instance, the reshaping of the digestive tract with the opening of the adult mouth and anus, the formation of Aristotle’s lantern, the growth of the endoskeleton, the maturation of the appendages already present, as well as the formation of new ones. At 8 dpm, the juveniles (Figure 16J) were hence bigger in size and more opaque to light than at 1 dpm (Figure 16A). More importantly, the juveniles were now grazing on the substrate, and were thus exotrophic. The initiation of grazing was the main landmark for the beginning of the *late juvenile stage*, which lasted for several months, until the reproductive organs were fully developed, and the animals started to produce gametes, marking the beginning of the *adult stage*. At 8 dpm, the juveniles had further a complete test. All of the initial skeletal plates (the genital, ocular, interambulacral, and ambulacral plates) were expanded and fused to one another. This generated a rigid shell protecting the inner organs, with the suture between the plates no longer being clearly observable (Figures 16J–L). On the oral side, the ambulacral and interambulacral plates were excluded from a zone free of endoskeleton, called the peristome (Formery et al., 2021), but in which the buccal plates developed (Figure 16J). At 8 dpm, the buccal plates were fully formed, and they created a protective skeletal structure below Aristotle’s lantern (Figures 16J,L). Yet, they remained independent of the other plates of the test to ensure the motility of the masticatory apparatus (Supplementary Video S7), which was now composed of five functional, pointed teeth protruding from the center of the oral surface (Figures 16J,L; Supplementary Video S7).

On the aboral surface of the 8 dpm juveniles, the pedicellariae inherited from the larva were still present, although they will eventually be replaced by stalked, adult pedicellariae (Gosselin and Jangoux, 1998). At 8 dpm, the juveniles were also characterized by new, round-tipped appendages on their lateral sides (Figures 16J,M). These were the so-called sphaeridia, which are believed to correspond to sensory organs involved in regulating the balance of the animal (Märkel et al., 1992). Regarding the podia, the primary podia were significantly reduced in size and exhibited less activity, corresponding only to protruding remnants at the level of the ocular plates (Figure 16N). In contrast, the ten initial secondary podia had considerably grown in size (Figures 16J,L) and were now the principal locomotor apparatus of the animal, along with other, additional secondary podia that formed following a stereotypical pattern of appearance referred to as the “baaba pattern” (Mooi et al., 2005; Morris, 2009). At their tip, all secondary podia had further a skeletal disk (Figure 16L), which, as opposed to the single spicule found in the skeletal disks of the primary podia (Figure 16G), were composed of three spicules that were fused together (Figure 16O). Finally, at 8 dpm, the definitive and juvenile spines inherited from the rudiment and the larva also reached their maximum length (Figures 16P,Q). The mature definitive spines had a single, pointed end resulting from the fusion of the six longitudinal processes constituting their stem, and they were motile by means of an articulated basal tubercle (Figure 16P). In contrast, the mature juvenile spines were multi-pointed, due to the outgrowth of their three or four longitudinal processes, and they were immotile (Figure 16Q).

## Discussion

The sea urchin *P. lividus* is one of the historical echinoid model species and, as such, has already been at the center of a large number of studies (e.g., Boveri, 1901; Hörstadius, 1939). Despite this fact, a rigorous developmental staging scheme from fertilization to juvenile stages, with details on coeloms and rudiment development, was still missing for this species. To fill this gap, we here propose an up-to-date staging scheme for the development of *P. lividus* from fertilization through metamorphosis to juvenile stages. Following the developmental trajectory of this animal in live specimens enabled us to recognize a total of 33 easily distinguishable stages divided into three main periods: embryonic, larval, and adult (Figure 3). In addition, we described 15 additional stages that specifically apply to the development of the coeloms and the rudiment and that take place independently during the larval period (see subsection in Figure 3). We further provide, for each of these 48 stages, key anatomical features that will enable both specialists and non-specialists to easily distinguish the different developmental stages using a simple compound microscope (Supplementary Figure S3). Taken together, this proposed developmental atlas compiles the fragmentary knowledge and existing descriptions of *P. lividus* development and adds new data on ciliogenesis, myogenesis, and development of the coeloms and the rudiment as well as on the process of metamorphosis. As such, it provides a wealth of high-resolution images and morphological descriptions to define the development of this species and to facilitate comparisons with other echinoids and beyond.

The study of echinoid development has led through the years to major breakthroughs in our understanding of cell biology, embryology, and gene regulation. However, unlike classical model systems, like the arthropod *Drosophila melanogaster* or the nematode *Caenorhabditis elegans*, studies on echinoid development have been carried out on different species, chiefly based on geographical availability (McClay, 2011; Ettensohn, 2017). This use of different echinoid species has been beneficial to provide a more accurate view of the conservation and divergence of genetic and developmental mechanisms within echinoids. Thus, although most species used in research laboratories are regular, indirect-developing echinoids, a number of species-specific traits have been identified, some of which proved challenging for establishing inter-species comparisons. In the following sections, we assess how our developmental staging scheme for *P. lividus* compares to that of other echinoid species and how it might be used to inform inter-species comparisons.

### Embryonic period

During the embryonic period, we retrieved the main developmental stages and features that were previously reported for *P. lividus* (e.g., Boveri, 1901; Hörstadius, 1973; von Ubisch, 1913), although for some stages or features we provided a more rigorous nomenclature and/or description.

A first example of a key feature that we retrieved, is the presence, in the egg, of a specific band of orange-pigmented granules, which had previously been described by several zoologists (e.g., Boveri, 1901; Sardet and Chang, 1985). This pigment band was located in the eggs in a subequatorial position and persisted throughout the cleavage period. This pigment band is a specificity of *P. lividus* and provides a considerable advantage for micromanipulation experiments, as it reveals the position of the first embryonic axis, the animal-vegetal axis, which is perpendicular to the band (Hörstadius, 1973; Croce et al., 2011).

Subsequently, during cleavage, blastula, and gastrula stages, we also found the same sequence of cell divisions and morphogenetic movements that had previously been described for *P. lividus* (e.g., Hörstadius, 1973; Robert et al., 2014). One important feature during these stages is the development of different types of cilia to enable propelling and directionality of the embryos and to ensure feeding and population dispersal (Metaxas, 2001; Chan et al., 2015). Using immunohistochemistry assays, we corroborated that cilia emerged in *P. lividus* embryos prior to hatching, when the embryo is composed of several hundred cells arranged as a spherical monolayer (Prulière et al., 2011). Yet, more specifically, we demonstrated that ciliogenesis started at the *mid-blastula stage*, as further corroborated by the rotating movement of the embryos within the fertilization envelope. Similarly, the apical tuft had previously been reported to emerge when the embryos began gastrulating (Prulière et al., 2011). Here, we sustained this finding, but established, more precisely, that the first long cilia, on the apical pole domain, appeared at the *mesenchyme blastula stage.*

Comparisons of the *P. lividus* data with those from other echinoid species, including some belonging to other echinoid groups (Supplementary Figure S4), revealed subtle differences during cleavage, blastula, and gastrula stages. For instance, although most echinoid species have a *16-cell stage* (e.g., this study, Hart, 1996; Vellutini and Migotto, 2010; Bennett et al., 2012; Dautov et al., 2020), the *28-* and *56-cell stages* we described for *P. lividus* have so far only been reported in a couple of camarodont and clypeasteroid species (Okazaki, 1975; Morrill and Marcus, 2005; Vellutini and Migotto, 2010; Nesbit and Hamdoun, 2020). In most other echinoids, the micromeres do not seem to divide later than the other embryonic cells, meaning that the *32-cell stage* directly follows the *16-cell stage* and directly precedes the *60-cell stage*, without intermediate *28-* or *56-cell stages*. Similarly, in at least one cidaroid (*Prionocidaris baculosa*), one diadematoid (*Centrostephanus rodgersii*), and one clypeasteroid (*Dendraster excentricus*), a *64-cell stage* was reported instead of a *60-cell stage* (Huggett et al., 2005; Olivares-Bañuelos et al., 2012; Yamazaki et al., 2012). In addition, ciliogenesis does not seem to take place at the same stage in all echinoids. For instance, in the camarodont *Lytechinus variegatus*, initiation of rotation within the fertilization envelope was reported as early as at the *early blastula stage*, and the first trace of an apical tuft was observed at hatching (Conway et al., 1984). Moreover, although gastrulation occurs in *P. lividus* as described in most other euechinoid species, it substantially differs from that reported in cidaroids, in which mesenchyme cells do not ingress prior to gut invagination (Schroeder, 1981; Bennett et al., 2012; Yamazaki et al., 2012). Other modifications to gastrulation compared to *P. lividus* have also been observed, for instance, in the camarodont *Colobocentrotus mertensii*, in which skeletogenesis is initiated prior to gut formation (Thet et al., 2004).

Despite these differences, one can reasonably consider that the embryonic period is very similar in most echinoid groups. As a matter of fact, the cleavage phase always includes holoblastic, meridional, and equatorial cleavages, which equivalently lead to the formation of a hollow sphere composed of a monolayer of cells surrounding an inner fluid-filled cavity. Following the cleavage period, the blastulae are also always ciliated prior to hatching, and hatching takes place prior to gastrulation. Likewise, the first elements of the larval skeleton always arise post-hatching, as two triradiate spicules within two bilateral cell clusters positioned in the vicinity of the vegetal pole. In all groups, gut formation also occurs by tissue invagination, and the gut elongates from the vegetal pole to the animal pole. Furthermore, in all echinoids studied so far, the embryonic period ends, at the *prism stage*, with the emergence of a triangular-shaped embryo lacking an opened mouth.

### Larval development

Regarding the larval period, we also identified the same main stages that had previously been reported for *P. lividus*, and which were named based on the development of the larval arms and the behavior of the larva (e.g., Gosselin and Jangoux, 1998; Paredes et al., 2015; von Ubisch, 1913). In *P. lividus*, the beginning of the larval period was marked by the opening of the mouth at the *early pluteus stage*, followed by the acquisition of the capacity to feed at the beginning of the *4-arm pluteus stage*. The larval period further featured the appearance and growth of 4 pairs of arms at the *2-*, *4-*, *6-*, and *8-arm pluteus stages*. Between the end of the *4-arm* and *8-arm pluteus stages*, the larval period was also characterized by the development of adult structures, and the larval period ended with competency, i.e., when the larval and adult structures were fully developed.

Although, in most echinoid species, the larval period is characterized by a similar set of features, including the opening of the mouth, feeding, the growth of arms, and the development of adult structures, there are marked differences between different echinoid groups. For instance, while in most indirect developing camarodonts and clypeasteroids the larva has eight arms by the end of the larval period, in diadematoids the fully developed larva only has two arms, in cidaroids eight arms and several vibratile lobes, and in spatangoids twelve arms and an apical process (e.g., Mortensen, 1921; 1931; Wray, 1992). Similarly, while in most echinoid species the opening of the mouth takes place at the onset of the larval period, in the cidaroid *Cidaris blakei*, the mouth only opens at the *2-arm pluteus stage* (Bennett et al., 2012). Moreover, in some species, such as the spatangoid *Echinocardium cordatum*, adult structures, like the hydropore, the vestibule, and the pedicellariae, do not form until the larva is fully developed (Nunes and Jangoux, 2007).

Another striking trait of larval development in *P. lividus* was the formation of the posterodorsal spicules, the dorsal arch, the pedicellariae, and the genial plates. In *P. lividus*, all these structures emerged from aggregates of mesenchyme cells of unknown origin. In the camarodonts *Pseudocentrotus depressus* and *Hemicentrotus pulcherrimus*, these structures have been suggested to derive from non-skeletogenic mesoderm cells delaminating, at the *late gastrula stage*, from the tip of the archenteron (Yajima, 2007). Whether this is also the case in *P. lividus* remains however to be demonstrated.

One additional major event taking place during the larval period in *P. lividus* was the establishment of the ciliary band and the epaulettes. We found that, in the *P. lividus* larva, the ciliated cells, within these structures, are organized into several rows and that a total of two pairs of epaulettes formed, two anteriorly and two posteriorly. The total number of epaulettes, their site of origin, their position, and their nomenclature actually vary between echinoid species (e.g., Wray, 1992). In the camarodonts *Echinus esculentus*, *Mesocentrotus nudus*, *Strongylocentrotus purpuratus*, and *H. pulcherrimus*, for instance, a total of six epaulettes form (organized in three pairs), referred to as either anterior and posterior or upper and lower epaulettes (MacBride, 1903; Mogami et al., 1991; Smith et al., 2008; Dautov et al., 2020). In *S. purpuratus* and *H. pulcherrimus*, the third pair of epaulettes arises as two buds from the ciliary band located, on each side of the larva, at the base of the posterodorsal and the postoral arm (i.e., from the lateral fields). These two buds subsequently migrate to a more dorsal (or lower) position than the two other pairs and eventually encircle the dorsal (or lower) end of the larva (Mogami et al., 1991; Smith et al., 2008). In *P. lividus*, we never observed a third pair of epaulettes, and the ciliary band always remained intact at the extremity of the lateral fields.

One last important characteristic of the larval period in *P. lividus* was the development of the larval musculature, for which we determined the ontogeny and organization. We showed that the *myosin heavy chain* (*mhc*) gene, an evolutionary conserved marker for the terminal differentiation of muscles (Wessel et al., 1990), was first expressed in *P. lividus* at the *prism stage* and that a more conspicuous musculature, surrounding the esophagus and allowing the larvae to feed, became detectable as early as at the beginning of the *4-arm pluteus stage*. In the camarodont species *S. purpuratus* and *H. pulcherrimus*, similar circumesophageal muscles, encircling the esophagus, have been described, and it has been suggested that these muscles form from muscle progenitor cells delaminating from the left and the right coelomic pouches (Ishimoda-Takagi et al., 1984; Burke and Alvarez, 1988). In *P. lividus*, we did not observe such a delamination, but the overall ontogeny of the circumesophageal muscles suggests that a similar process might exist in this species as well. Contrasting with studies in other echinoids (e.g., Strathmann, 1971; Burke and Alvarez, 1988; Dyachuk and Odintsova, 2013; MacNeil et al., 2017), we did not find, in *P. lividus*, the basal ring muscles reported in the camarodont *L. variegatus* and the cidaroid *Eucidaris tribuloides* (MacNeil et al., 2017). However, our work revealed, in *P. lividus* larvae, two additional muscular structures: the preoral dilator muscles, extending in the preoral arms, and the mesenteries, surrounding the larval digestive tract. The preoral dilator muscles, like the anterior dilator muscles, are positioned in the arms borne by the oral hood. We thus propose that, similar to the anterior dilator muscles (Strathmann, 1971), the preoral dilator muscles are involved in widening the mouth aperture and bending the arms to optimize food catching. The mesenteries appeared shortly before the larva reached competency and were chiefly surrounding the larval stomach. Although the roles of the mesenteries remain unknown, they are likely to persist in the juvenile (Formery et al., 2021), which also inherits the larval stomach (Fenaux et al., 1994).

### Coeloms and adult rudiment development

Despite the wealth of developmental and anatomical studies on *P. lividus*, and the importance of the coeloms and the rudiment for the formation of the adult in indirect-developing echinoids, detailed staging schemes covering the development of the coeloms and the rudiment in echinoids have so far only been compiled for two camarodont species, *H. pulcherrimus* and *S. purpuratus* (Chino et al., 1994; Smith et al., 2008; Heyland and Hodin, 2014). In these two camarodonts, as in *P. lividus*, development of the coeloms and the rudiment takes place in the feeding larva, in trajectories that are independent of that of the rest of the larva. The staging scheme we propose here for *P. lividus* is largely similar to that of the other two camarodonts, following the logic of the developing anatomical landmarks. For instance, in *S. purpuratus*, as in *P. lividus*, the development of the coeloms starts with the projection of the hydroporic canal and the formation of the hydropore, and hence by an equivalent *hydroporic canal stage* (Smith et al., 2008). Thereafter, the vestibule invaginates and contacts the left hydrocoel, the pentaradial symmetry of the adult emerges at the level of the hydrocoel, and the primary podia appear (Chino et al., 1994; Smith et al., 2008; Heyland and Hodin, 2014). In all three camarodont species, the development of the rudiment also then ends at a *mature rudiment stage* with a fully developed rudiment characterized by five primary podia, a number of juvenile and definitive spines, pedicellariae, and genital plates (Chino et al., 1994; Smith et al., 2008; Heyland and Hodin, 2014). Despite these similarities, we defined two additional stages in *P. lividus* corresponding to the twisting of the primary podia and the emergence of the juvenile spines. These events are also likely to occur in the two other camarodont species, *S. purpuratus* and *H. pulcherrimus*, but in these species they have not yet been recognized as individual stages. We were further able to observe the circularization of the left hydrocoel leading to the establishment of the ring canal at the *5-fold mesoderm stage*, an event that has so far only been documented by von Ubisch (1913).

Apart from these species, relatively little is known about the development of the coeloms and the rudiment in other echinoids. It has been shown, for instance, that in the cidaroids *Eucidaris thouarsi* (Emlet, 1988) and *C. blakei* (Bennett et al., 2012), the clypeasteroid *Clypeaster subdepressus* (Vellutini and Migotto, 2010), and the spatangoid *E. cordatum* (Gordon, 1926; Nunes and Jangoux, 2007) a rudiment also forms after the larva starts feeding, on the left side of the digestive tract, at the level of the stomach. These echinoids also feature a *mature rudiment stage* characterized by a fully developed adult rudiment with 5 primary podia and several spines. The type of spines formed at the level of the rudiment, their number as well as the timing of their formation, however, vary between species (e.g., Emlet, 1988; Huggett et al., 2005; Nunes and Jangoux, 2007; Vellutini and Migotto, 2010; Bennett et al., 2012; Rahman et al., 2012; Rahman et al., 2015). Other differences observed between echinoid species concern the morphological changes leading to the emergence of the rudiment. In the camarodont Temnopleurid species, *Temnopleurus toreumaticus* and *Temnopleurus reevesii*, for instance, the rudiment does not form by invagination of the larval epidermis as in *P. lividus*, *S. purpuratus*, and *H. pulcherrimus*. In these Temnopleurid species, instead, an inner cell mass forms during the larval stages, and the adult rudiment subsequently develops from this inner cell mass (Kitazawa et al., 2014). Similarly, in cidaroids, development of the rudiment does not involve the emergence of a vestibule and the establishment of a vestibular cavity. Instead, in this echinoid group, the rudiment develops directly by embedding and folding of the larval epidermis onto the left hydrocoel (Emlet, 1988; Bennett et al., 2012). Given that the cidaroids are the earliest-branching group of echinoids (Supplementary Figure S3) (Thompson et al., 2017; Mongiardino Koch et al., 2018; Lin et al., 2020; Horton et al., 2022), studying development of the coeloms and the rudiment in this group hence has the potential to provide important insights into the evolution of this process in echinoids.

### Competency and metamorphosis

In *P. lividus*, the end of the larval period was marked by the *competent pluteus stage* and by metamorphosis, i.e., the transformation of the larva into a benthic juvenile. In previous reports, competency of the larva to undergo metamorphosis has been defined either by the appearance of novel larval behaviors (Strathmann, 1978), by using metamorphosis-inducing pharmacological agents (e.g., Nunes and Jangoux, 2007), or by the protrusion of primary podia through the vestibular pore (Smith et al., 2008). In our hands, metamorphosis was inducible using dibromomethane treatments (Agatsuma et al., 2006; Taris et al., 2010) even in premature larvae, leading to non-viable juveniles. We thus propose to refer to the *competent pluteus stage* as: 1) the stage at which the larva has completed its development and the rudiment has reached the *mature rudiment stage*, and 2) the stage at which the larva starts exhibiting new behaviors, such as reduced swimming and probing the environment with primary podia protruding through the vestibular pore. This definition is coherent with what has been reported both in other camarodont species and in other echinoids, including the cidaroids, diadematoids, clypeasteroids, and spatangoids (e.g., Emlet, 1988; Nunes and Jangoux, 2007; Vellutini and Migotto, 2010; Rahman et al., 2015; Nesbit and Hamdoun, 2020).

In *P. lividus*, the process of metamorphosis has previously been described using scanning electron microscopy (Gosselin and Jangoux, 1998). Here, we used live recordings of metamorphosis to obtain a better resolution of the sequence of events defining this process. We thus identified two main steps of metamorphosis: 1) the opening of the vestibular pore, concomitant with the retraction of the epidermis of the larval arms along the skeletal rods and the folding of the epidermis of the larval body, and 2) the delamination of cells on the ventral side of the larva, concomitant with the smoothening of the epidermis. These two steps led to the emergence of the benthic, pentaradial juvenile, an observation that is once again consistent with previous descriptions of metamorphosis in other echinoids (e.g., Emlet, 1988; Nunes and Jangoux, 2007; Vellutini and Migotto, 2010; Rahman et al., 2015; Nesbit and Hamdoun, 2020). However, differences exist between echinoid species. For instance, due to the lack of vestibule formation, cidaroids also lack a vestibular pore (Emlet, 1988; Bennett et al., 2012). Also, in the camarodont species *C. mertensii*, resorption at the level of the larval arms concerns both the larval arm epithelium and the endoskeleton (Thet et al., 2004), while in *P. lividus* as well as in other camarodont, diadematoid, and clypeasteroid species it only affects the larval arm epithelium (Huggett et al., 2005; Vellutini and Migotto, 2010).

Gosselin and Jangoux (1998) reported that, during metamorphosis, parts of the larval epidermis, such as the epidermis associated with the genital plates and their appendages, merged with the everted vestibular lip and walls. As we could not confirm this observation, additional experiments, involving, for instance, cell tracking analyses, will be required to assess the fate of the larval epidermis during and after metamorphosis in *P. lividus*. More generally, it will be important to determine, which portions of larval tissues, including at the level of the digestive tract and the apex, are inherited by the juvenile upon metamorphosis. Likewise, future research should determine the origin of the cells that delaminate at the time of metamorphosis. Furthermore, previous studies investigating the role of programmed cell death in echinoid larva undergoing metamorphosis have concluded that apoptosis was chiefly limited to the larval arms during resorption of the epidermis (Roccheri et al., 2002; Sato et al., 2006). However, given the importance of programmed cell death for tissue remodeling during metamorphosis in a number of different animals, including frogs, insects, phoronids, mollusks, and hemichordates (Gifondorwa and Leise, 2006; Heyland and Moroz, 2006; Temereva and Tsitrin, 2014; Bump et al., 2022), additional analyses of this process in echinoids, such as in *P. lividus*, might be warranted. They should reveal novel information on the involvement of programmed cell death in the transition from larval to adult life (Heyland and Moroz, 2006; Fuchs and Steller, 2011) and should thus contribute to a more general understanding of the evolution of animal metamorphosis (Heyland and Hodin, 2014; Holstein and Laudet, 2014).

### Adult period

Following metamorphosis, the *P. lividus* juvenile had a spherical body with different appendages. Although its development was initially endotrophic, it soon started grazing on algae (i.e., about 8 days post-metamorphosis), thereby growing in size and eventually developing reproductive capabilities. Three major phases characterize the adult life of an echinoid, and this despite the echinoid group (Gosselin and Jangoux, 1998; Nunes and Jangoux, 2007; Vellutini and Migotto, 2010; Rahman et al., 2015; Nesbit and Hamdoun, 2020). The first phase is the endotrophic phase, which immediately follows metamorphosis (i.e., the *early juvenile stage*). The mouth and anus of the juvenile are still closed and the digestive tract is not yet functional. The second phase starts when the juvenile becomes exotrophic (i.e., the *late juvenile stage*). The digestive tract is now functional and the animal grazes on algae, although it is not yet sexually mature. The third phase begins when the animal becomes sexually mature (i.e., the *adult stage*). It thus possesses gonads and produces gametes.

In *P. lividus*, previous work has already reported on the anatomy of the *early* and *late juvenile stages*, using essentially immunohistochemistry and scanning electron microscopy (Gosselin and Jangoux, 1998; Formery et al., 2021; Thompson et al., 2021). Here, we corroborated the previously described structural features defining these two developmental stages. Our work thus lends further support to the observation that newly-metamorphosed juveniles of echinoids are characterized by a highly conserved morphology, in the form of a spherical body with different types of appendages, and this even in species with radically divergent adult anatomies, such as in irregular echinoids (e.g., Gordon, 1929; Okazaki and Dan, 1954; Emlet, 1988). However, from one species to another, these appendages differ in number and type. There might be different numbers and types of spines, for instance, as well as more or fewer podia, pedicellariae, and sphaeridia (Gosselin and Jangoux, 1998; Emlet, 1999; Nunes and Jangoux, 2007). As an example, following metamorphosis, a *P. lividus* juvenile always has five primary podia, twenty definitive spines, fifteen to nineteen juvenile spines, and zero to four pedicellariae. In contrast, at the same stage, the clypeasteroid *C. subdepressus* has no pedicellariae (Vellutini and Migotto, 2010) and the cidaroid *C. blakei* has one to three pedicellariae along with five to twenty-three spines of the same type (Bennett et al., 2012).

## Conclusion

Taken together, this study represents a comprehensive description of *P. lividus* development, providing a complete compendium of its embryonic, larval, and adult periods. This dataset thus represents a useful framework for future research on this species as well as for future comparisons with other animals, echinoids, echinoderms, and beyond. Our results will also be useful for developing reliable rearing conditions for echinoid aquaculture, as they provide easily recognizable characteristics to identify specific developmental stages during different periods of the life cycle. With the advancement of genome editing techniques in marine organisms and the relatively short generation time of *P. lividus*, this work also creates a solid basis for the creation of stable transgenic lines in this echinoid species, opening the door for genetic approaches in this animal model system.

## Data Availability

The original contributions presented in the study are included in the article/Supplementary Material, further inquiries can be directed to the corresponding author.
