# Supplementary material for: Developmental atlas of the indirect-developing sea urchin Paracentrotus lividus: From fertilization to juvenile stages
Source: Front Cell Dev Biol. 2022 Oct 31;10:966408. doi: 10.3389/fcell.2022.966408 (PMC9664562; doi:10.3389/fcell.2022.966408)
Supplement: Supplementary file 3 [file DataSheet1.pdf]

SUPPLEMENTARY MATERIAL

Supplementary Figures

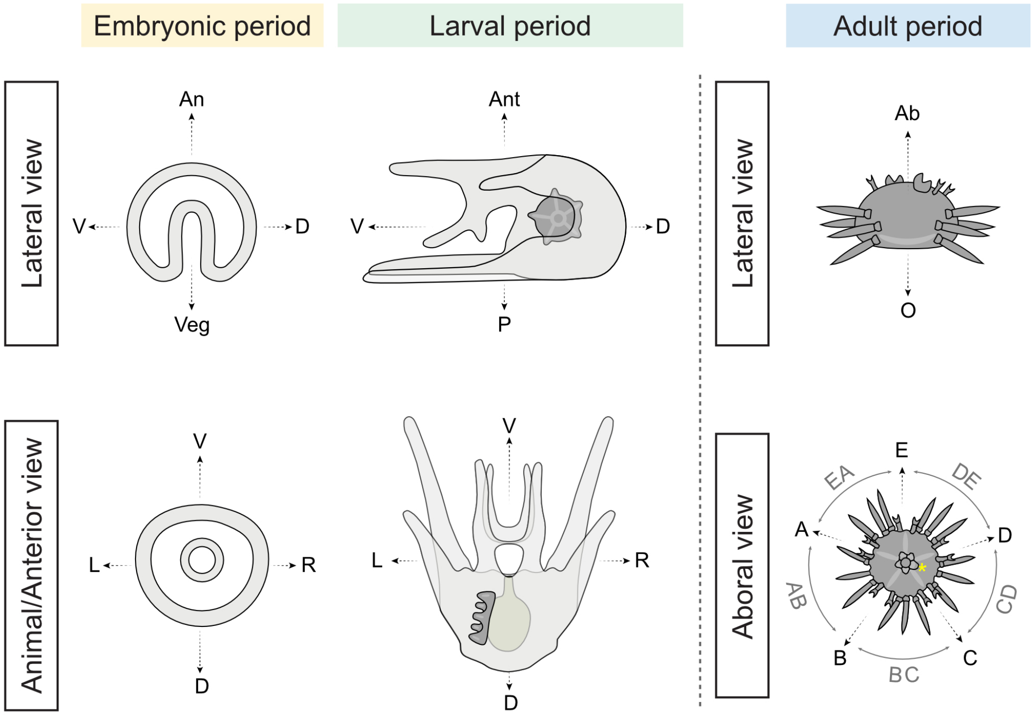

Supplementary Figure S1. **Nomenclature of embryonic, larval, and juvenile axes**. Schematic representations of the terminology used to refer to the developmental axes of the embryos, larvae, and juveniles of *Paracentrotus lividus*. The embryonic period is represented by a mid-gastrula stage, the larval period by an 8-arm pluteus stage bearing a rudiment at the primary podia stage, and the adult period by an early juvenile stage. In the rudiment and the juvenile, the water vascular system is highlighted in light gray. In the juvenile, the pentaradial symmetry of the body plan is shown according to Carpenter's nomenclature (1884). Therefore, A, B, C, D, E correspond to the five axes delimitated by the ambulacral fields, and AB, BC, CD, DE, EA correspond to the five interambulacral fields. Of note, the axes are indicated on the juvenile, in aboral view, using a counterclockwise Carpenter's nomenclature, as the original clockwise Carpenter's nomenclature was defined from observing the adult from the oral side (Carpenter, 1884; Paul and Hotchkiss, 2020). In the aboral view of the juvenile, the central disk of the aboral surface is further indicated: it is composed of 4 genital plates plus the madreporite (marked by a yellow asterisk). Ab: aboral; An: animal; Ant: anterior; D: dorsal; L: left; O: oral; P: posterior; R: right; V: ventral; Veg: vegetal.

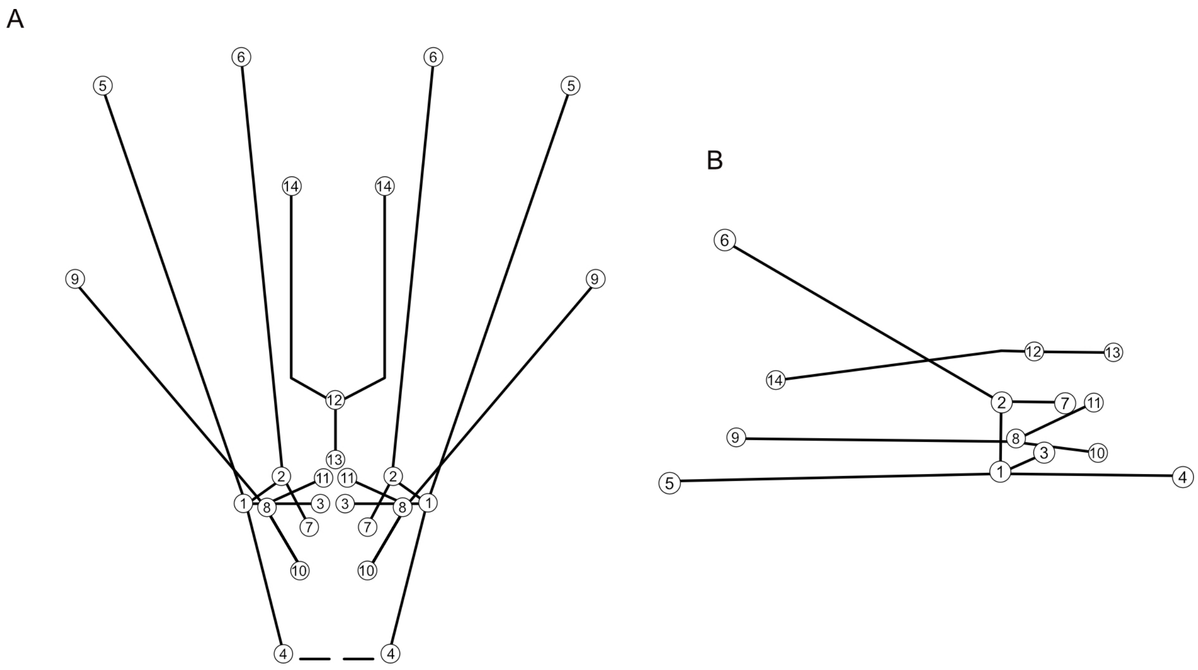

Supplementary Figure S2. **Larval skeletal rod nomenclature**. **(A, B)** Schematic representations of the skeletal rods of an 8-arm pluteus larva in anterior view, with the ventral side up and the left side to the left in **(A)**, and in lateral view, with the ventral side to the left and the anterior side up in **(B)**. Each rod is identified by two numbers marking its extremities. (1-2) dorsoventral connecting rods, (1-3) ventral transverse rods, (1-4) body rods, (1-5) postoral rods, (2-6) anterolateral rods, (2-7) recurrent rods, (8-9) posterodorsal rods, (8-10) anteroventral rods, (8-11) anterodorsal rods, (12-13) anteromedial rod, and (12-14) preoral rods.

Period		Stage	Defining characters
Embryonic	Cleavage	1-cell stage (zygote)	The embryo has one cell
		2-cell stage	The embryo has two cells
		4-cell stage	The embryo has four cells
		8-cell stage	The embryo has eight cells
		16-cell stage	The embryo has sixteen cells
		28-cell stage	The embryo has twenty-eight cells
		32-cell stage	The embryo has thirty-two cells
		56-cell stage	The embryo has fifty-six cells
		60-cell stage	The embryo has sixty cells
	Blastula	very early blastula stage	Cell tiers and embryonic axes are no longer distinguishable; the sole usable landmark is the timing of development after the 60-cell stage (i.e., 1 hour)
		early blastula stage	No specific morphological character; the sole usable landmark is the timing of development after the 60-cell stage (i.e., 2 hours)
		mid-blastula stage	No specific morphological character; the sole usable landmark is the timing of development after the 60-cell stage (i.e., 3 hours)
		late blastula stage	No specific morphological character; the sole usable landmark is the timing of development after the 60-cell stage (i.e., 4 hours)
		hatched blastula stage	The embryo hatches from the fertilization envelope
		swimming blastula stage	The vegetal pole thickens
		late swimming blastula stage	The vegetal pole exhibits a "V" shape
	Gastrulation	early mesenchyme blastula stage	The skeletogenic mesoderm cells start to ingress
		late mesenchyme blastula stage	All skeletogenic mesoderm cells have ingressed
		blastopore formation stage	The blastopore becomes visible
		early gastrula stage	The archenteron is about one-third into the blastocoel; the lateral chains are formed
		mid-gastrula stage	The archenteron is half-way into the blastocoel; cells at the tip of the archenteron delaminate
		late gastrula stage	The archenteron reaches the blastocoel roof
		prism stage	The embryo has a triangular shape
Larval		early pluteus stage	The mouth opens
		2-arm pluteus stage	The postoral arms are apparent
		4-arm pluteus stage	The anterolateral arms are apparent
		6-arm pluteus stage	The posterodorsal arms are apparent
		8-arm pluteus stage	The preoral arms are apparent
		competent pluteus stage	The vestibular pore is open; the larval arm epidermis has started to shrink; epaulette beating is reduced; one primary podium protrudes outside the vestibular pore
		metamorphosis	Metamorphosis is triggered
Adult		early juvenile stage	The juvenile does not have an open mouth
		late juvenile stage	The juvenile has a functional digestive tract, but no reproductive organs
		adult stage	Sexual maturity is reached

Coeloms and Adult rudiment stage	Defining characters
hydroporic canal stage	The hydroporic canal is formed
constricted coelomic pouch stage	Constrictions are present in the elongated coelomic pouches
stone canal stage	The stone canal is apparent
vestibule invagination stage	The vestibule starts to invaginate
vestibule contact stage	The vestibular floor makes contact with the left hydrocoel
vestibule flattened stage	The vestibular floor is flat
vestibule waving stage	The apical surface of the vestibular floor is exhibiting folds; the left hydrocoel displays a pentaradial symmetry
5-fold mesoderm stage	The left hydrocoel is folded, but not the basal surface of the vestibular floor
5-fold ectoderm stage	The left hydrocoel and the basal surface of the vestibular floor are folded
primary podia stage	The primary podia are apparent
primary podia touching stage	The tips of the primary podia touch each other
primary podia twisting stage	The primary podia twist within the vestibular cavity
primary podia papilla stage	The papilla at the tip of the primary podia is formed
juvenile spine stage	The juvenile spines have emerged
mature rudiment stage	The adult rudiment is larger than the larval stomach; the juvenile spines are about half the length of the definitive spines

Supplementary Figure S3. Defining characters for the *Paracentrotus lividus* staging scheme. As in Figure 3, since development of the coeloms and the adult rudiment proceeded in parallel to that of the larva, the events related to the development of the coeloms and the adult rudiment are provided in the subsection in the right panel, in parallel to the 4-, 6-, and 8-arm pluteus stages.

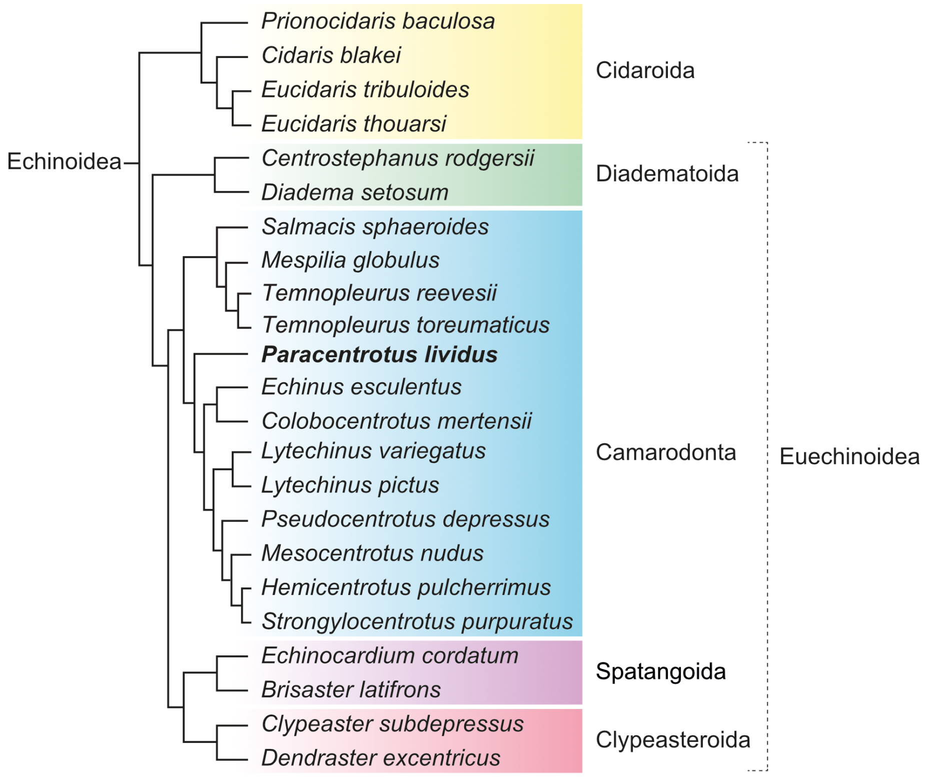

Supplementary Figure S4. Phylogenetic relationship of echinoid species and groups referred to in the discussion. The tree is based on Mongiardino Koch et al., 2018, Lin et al., 2020, and the World Register of Marine Species (WoRMS) (Horton et al., 2022). The cidaroids form an independent clade and are the sister group of all other echinoids, which are called the euechinoids.

Supplementary Videos

Supplementary Video S1. Larval swallowing. Movie showing the muscular movements at the level of the esophagus and the cardiac sphincter in a 4-arm pluteus stage larva shown in lateral view. Cs: cardiac sphincter; Es: esophagus.

Supplementary Video S2. Axocoel pumping. Movie showing the activity of the left axocoel in a larva bearing an adult rudiment at the primary podia papilla stage in anterior view. The black arrow marks the left axocoel. Es: esophagus; St: stomach.

Supplementary Video S3. Circulating coelomocytes. Movie showing coelomocytes moving back and forth in the ring and radial canals of the water vascular system in a larva bearing an adult rudiment at the primary podia papilla stage in left, deep view.

Supplementary Video S4. Podia motility. Movie showing the motility of the primary podia within the vestibular cavity in a larva bearing an adult rudiment at the primary podia papilla stage in left, deep view.

Supplementary Video S5. **Pedicellaria opening and closing movement**. Movie showing the opening and closing movements of the three jaws of a pedicellaria on the aboral surface of a larva at the 8-arm pluteus stage.

Supplementary Video S6. *Paracentrotus lividus* metamorphosis. Movie showing a *Paracentrotus lividus* larva undergoing metamorphosis. The ventral side of the larva, marked by the position of the larval arms, is directed towards the bottom left corner. The vestibular pore is on the opposite side.

Supplementary Video S7. **Aristotle's lantern motility**. Movie showing the motility of Aristotle's lantern and of the teeth in an 8-day post-metamorphic juvenile in oral view.

Supplementary References

Carpenter, P. H. (1884). Report upon the Crinoidea collected during the voyage of H.M.S. Challenger during the years 1873-76. Part I: General morphology, with descriptions of the stalked crinoids. *Rep. Sci. Results Explor. Voyage H.M.S. Challenger, Zoology* 11, 1–442.

Horton, T., Kroh, A., Ahyong, S., Bailly, N., Bieler, R., Boyko, C. B., et al. (2022). World Register of Marine Species (WoRMS). Available at: https://www.marinespecies.org (Accessed 04 2022, 20).

Lin, J.-P., Tsai, M.-H., Kroh, A., Trautman, A., Machado, D. J., Chang, L.-Y., et al. (2020). The first complete mitochondrial genome of the sand dollar *Sinaechinocyamus mai* (Echinoidea: Clypeasteroida). *Genomics* 112, 1686–1693. doi:10.1016/j.ygeno.2019.10.007

Mongiardino Koch, N., Coppard, S. E., Lessios, H. A., Briggs, D. E. G., Mooi, R., and Rouse, G. W. (2018). A phylogenomic resolution of the sea urchin tree of life. *BMC Evol. Biol.* 18, 189. doi: 10.1186/s12862-018-1300-4.

Paul, C. R. C., and Hotchkiss, F. H. C. (2020). Origin and significance of Lovén's Law in echinoderms. *J. Paleontol.* 94, 1089–1102. doi: 10.1017/jpa.2020.31.
